# METTL3-mediated SNHG1 m^6^A modification promotes proliferation and migration through transcriptional regulation of WDR74 in osteosarcoma

**DOI:** 10.3389/fonc.2025.1529657

**Published:** 2025-05-29

**Authors:** Guanzhen Qiu, Yuxin Bao, Yuanzhuang Zhang, Yeqiu Xu, Tianhua Qiao, Chenghao Li, Hanjie Zhai, Zhenjun Chen, Fu Ren, Yong Wang

**Affiliations:** ^1^ Second Department of Spine Surgery, Central Hospital Affiliated to Shenyang Medical College, Shenyang, Liaoning, China; ^2^ Department of Neurosurgery, Central Hospital Affiliated to Shenyang Medical College, Shenyang, Liaoning, China; ^3^ Department of Anatomy, School of Basic Medicine, Shenyang Medical College, Shenyang, Liaoning, China

**Keywords:** METTL3, N6-methyladenosine, SNHG1, WDR74, osteosarcoma, proliferation/metastasis

## Abstract

**Introduction:**

As the most prevalent internal RNA modification in eukaryotic transcripts, N6-methyladenosine (m^6^A) which is catalyzed by methyltransferase-like 3 (METTL3), is widely involved in cancerous diseases. However, the role of METTL3 and small nucleolar RNA host gene 1 (SNHG1) playing in osteosarcoma (OS) remains largely unknown.

**Methods:**

Bioinformatics analysis, RT-qPCR, western blotting assays were used to detect the expression of METTL3, SNHG1, RNA binding motif protein 15 (RBM15), WD repeat domain 74 (WDR74) and EWS RNA binding protein 1 (EWSR1) accordingly. Cell proliferation and motility ability changes were assessed by colony formation and transwell migration assays. RNA stability changes were evaluated by an *actinomycin* D assay. The level of SNHG1 m6A modification changes were addressed by an RNA immunoprecipitation (MeRIP)-qPCR assay. RNA pulldown assays and RNA immunoprecipitation assays were applied to detect the interactions between SNHG1 and proteins. A chromatin immunoprecipitation (ChIP)-qPCR assay was performed to verify the binding effect between WDR74 promoter region and EWSR1. Orthotopic xenograft mouse models were constructed to evaluate the role of METTL3 playing in OS tumorigenesis and lung metastasis *in vivo*.

**Results:**

It was uncovered that METTL3 was significantly upregulated in OS tissues and cell lines. As an oncogenic regulator, METTL3 promoted proliferation and migration in OS cells by enhancing the stability of SNHG1. Mechanically, it was displayed that METTL3 catalyzed SNHG1 m6A modification with the assistance of RBM15. More deeply, it was found that SNHG1 promoted OS cells proliferation and migration via regulation of its neighboring gene WDR74. Meanwhile, it was discovered that SNHG1 affected WDR74 transcription by EWSR1 recruitment. Finally, it was displayed that overexpression of METTL3 promoted SNHG1 and WDR74 expression, and upregulation of METTL3 facilitated OS tumorigenesis and lung metastasis *in vivo*.

**Conclusion:**

The present research illustrated that METTL3 enhanced the stability of SNHG1 with the assistance of RBM15 in an m6A dependent manner in OS cells. And SNHG1, promoted the transcription of WDR74 in cis, via recruitment of EWSR1, thereby facilitated WDR74-mediated proliferation and migration in OS cells. These findings provide new insights into the epigenetic regulation of OS and highlight potential therapeutic targets.

## Introduction

Osteosarcoma (OS), though a rare primary bone tumor of the musculoskeletal system, accounts for approximately 20% of all primary bone malignancies, with an estimated global incidence of 30,000 cases annually (1). Due to its aggressive clinicopathological features, the overall prognosis of OS remains poor, with a 5-year survival rate of only 61.6% in young-onset patients (2). Although the combination of adjuvant chemotherapy and surgical resection has significantly improved the survival rate, the event-free survival rate in patients with metastasis remains below 30% (3). Consequently, a comprehensive understanding of the molecular biology of OS and the identification of novel targets remain critical in the treatment of OS.

m^6^A, catalyzed by methyltransferase complexes (MTCs) and characterized by the consensus motif RRACH (where *R* = *A*/*G*, *H* = *A*/*C*/*U*), is the most prevalent posttranscriptional modification on RNAs, including both mRNAs and ncRNAs (4). METTL3, with a length of 580 amino acids, a key component of MTCs, is widely involved in cancer progression and therapeutic targeting (5). METTL3 acts as an m^6^A methyltransferase and functions as both an oncogenic regulator and a tumor suppressor in diverse cancers (5). With the assistance of certain readers, such as insulin-like growth factor 2 mRNA-binding protein 2 (IGF2BP2), insulin-like growth factor 2 mRNA-binding protein 3 (IGF2BP3), and YTH N6-methyladenosine RNA-binding protein F1 (YTHDF1), METTL3-mediated RNA methylation adjusted the RNA stability in an m^6^A-dependent manner (6–8). METTL3 also increased the stability of multiple lncRNAs like MALAT1, THAP domain containing seven antisense RNA 1 (THAP7-AS1), small nucleolar RNA host gene 7 (SNHG7) and promotes various cancers progression including glioma, gastric cancer and prostate cancer (9–11). To date, related research on METTL3 and lncRNA in OS is rare. It was reported by Zhou et al. that METTL3 increased differentiation antagonizing non-protein coding RNA (DANCR) stability via m^6^A modification and contributed to OS progression (12). However, whether METTL3 might regulate SNHG1 remains unclear in OS.

SNHG1 is localized at the chromosome 11q12.3 region and contains 11 exons. SNHG1 is well recognized as an oncogene in diverse cancers (13, 14). SNHG1 exerts oncogenic roles via absorbing multiple miRNAs like miR-577, miR-493-3p, miR-326, miR-101-3p, and miR-424-5p through acting as a competing endogenous RNA (ceRNA) in OS (15–19). LncRNAs regulate downstream gene expression at different levels through myriad mechanisms, including affecting RNA splicing, regulating transcription of neighboring and distant genes, and adjusting RNA stability and translation by interacting with DNA, RNA, and proteins (20). SNHG1 is upregulated and is mainly localized in the nucleus of gastric cancer, colorectal cancer, liver cancer, and lung cancers (21–24). Mechanically, it was displayed that SNHG1 affected focal adhesion kinase (FAK)/PI3K/AKT signaling pathway by regulation of its neighboring gene solute carrier family 3 member 2 (SLC3A2) (25). Unlike previous research, in the current study, it was found that SNHG1, methylated and stabilized by METTL3-initiated m^6^A, promoted OS cell proliferation and migration via regulation of its neighboring gene WD repeat domain 74 (WDR74). The current research unveiled a novel angle of illustrating how SNHG1 works in OS.

## Materials and methods

### Patients and tissue samples

OS tissue specimens and adjacent nontumor tissue specimens (5 cm away from the tumor) were collected from patients with OS according to a definite pathological diagnosis at Central Hospital Affiliated with Shenyang Medical College (Shenyang, China) and at Shengjing Hospital of China Medical University (Shenyang, China) during surgical resections. All patients have been informed and consented to be involved in this study. Permission for this study was granted by the Institute Research Medical Ethics Committee of Shenyang Medical College.

### Bioinformatics analysis and software availability

The differentially expressed data of m^6^A-related genes, SNHG1, and WDR74 OS-related GEO datasets GSE12865, GSE42352, GSE87437, and GSE33458 were downloaded from the GEO database and reanalyzed according to various analytical demands. The expression levels of METTL3 and WDR74 in TCGA database were analyzed using an online web tool, UALCAN (26). The survival analyses of METTL3, SNHG1, and EWSR1 levels in OS were performed using TCGA database (TARGET-OS) from an online web tool, PCAT (http://www.pedtranscriptome.org./?analysis).

### Cell culture

The human osteoblast cell line hFOB 1.19 was maintained in DMEM/F12 (Gibco, El Paso, TX, USA). Four human OS cell lines—MG-63, HOS, U2OS, and 143B—purchased from the Cell Bank of the Chinese Academy of Sciences (Shanghai, China), were cultured in DMEM (Gibco). All cells were incubated in a humidified atmosphere containing 5% CO_2_ at 37°C. All media were supplemented with 10% (v/v) fetal bovine serum (FBS; Sigma, St. Louis, MO, USA), 100 IU/mL penicillin (Baomanbio, Shanghai, China), and 100 mg/mL streptomycin (Baomanbio, China).

### Cell transfection and Oligo RNA transfection

The short hairpin RNAs (shRNAs) overexpression plasmids targeted or carried diverse genes, including METTL3 (shMETTL3-1, shMETTL3-2, and oeMETTL3), RBM15 (shRBM15–1 and shRBM15-2), SNHG1 (shSNHG1-1, shSNHG1-2, and oeSNHG1), and EWSR1 (shEWSR1-1, shEWSR1-2, and oeEWSR1) were designed and synthesized by Shanghai GenePharma Co. Ltd. (Shanghai, China). When OS cells were grown to 70% confluence, the shRNAs or overexpression plasmids were transfected into OS cells by using a Lipofectamine 3000 kit (Invitrogen, Carlsbad, CA, USA) according to the manufacturer’s instructions.

### RNA extraction and quantitative real-time PCR

The procedure was conducted as previously described (27). A TRIzol™ Plus RNA Purification Kit (Invitrogen) was used to isolate total RNAs from tissue specimens or cells. The nuclear and cytoplasmic RNAs were extracted by using a Cytoplastic and Nuclear RNA Purification Kit (Norgen BioTek, Thorold, Canada) according to the manufacturer’s protocol. Two micrograms of isolated RNAs were reversely transcribed into cDNAs using a PrimeScript™ RT Master Mix reagent kit (TaKaRa, Beijing, China). A TB Green^®^ Premix Ex Taq™ II reagent kit (TaKaRa) was used to perform the following RNA extraction and quantitative real-time PCR (RT-qPCR) assays according to the manufacturer’s instructions. GAPDH was set as an internal control, and the relative expression levels of target genes were calculated using the 2^−ΔΔCt^ method. All the primers were synthesized by TaKaRa, and the sequences are listed in **Supplementary Table S1**.

### Western blot analysis

The procedure was carried out as previously described (28). In brief, total proteins from cells or tissue samples were harvested by a protein extraction kit (Servicebio, Wuhan, China) and qualified by a BCA protein assay kit (Servicebio). Protein samples (20 μg) were separated by 10% sodium dodecyl sulfonate polyacrylamide gel electrophoresis (SDS-PAGE), transferred onto a PVDF membrane (Millipore, Billerica, MA, USA), and blocked for 1 h at room temperature. The membrane was sealed and then incubated with primary antibodies overnight at 4°C, individually. The next day, the primary antibodies were washed out, and the membrane was incubated with a second antibody at room temperature for 1 h. Lastly, the membrane was stained using a Hypersensitivity ECL Chemiluminescence Detection Kit (Sevenbio, Beijing, China). Protein signals were exposed by a gel imager (ChemiScope6100, Clinx Science Instruments Co. Ltd., Shanghai, China). Antibodies were as follows: METTL3 (1:5,000, Abcam, Cambridge, MA, UK, No. ab195352), RBM15 (1:2,000, Abcam, No. ab70549), WDR74 (1:1,000, Abcam, No. ab154190), GAPDH (1:10,000, Abcam, No. ab8245), Tubulin (1:5,000, Abcam, No. ab7291).

### Transwell assay

The procedure was performed as previously reported (29). HOS and U2OS cells with different interventions (with a density of 4 × 10^4^ for migration and 8 × 10^4^ for invasion assay) were incubated in the upper chambers (Corning, New York, USA). Medium without FBS was added to the upper chambers, while medium containing 10% FBS was added to the lower chambers, respectively. After 18 h, nonmigrated or noninvaded cells were wiped out, while migrated HOS and U2OS cells were fixed, stained, and counted using an inverted microscope (Olympus, Tokyo, Japan).

### Colony formation assay

HOS and U2OS cells with different interventions were seeded in a six-well plate with a density of 500 cells/well. The cells were supplemented with a culture medium containing 10% FBS and incubated with a condition of 5% CO_2_ at 37°C. After 10–14 days, the cells were fixed with 4% formalin and stained with crystal violet, and the formed colonies were counted.

### Actinomycin D assay

The procedure was performed as previously described (30). HOS and U2OS cells with different interventions were seeded into the six-well plate with a density of 2 × 10^5^ and then exposed to 2 μg/mL actinomycin D (Merck KGaA, Darmstadt, Germany) at different time points. Total RNAs from diverse cells at different time points were extracted, and the expression of SNHG1 was determined by an RT-qPCR assay.

### Methylated RNA immunoprecipitation qPCR assay

The procedures were performed according to the manufacturer’s instructions for a Magna MeRIP™ m^6^A Kit (Merck KGaA). In brief, isolated total RNAs from tissue specimens or cells were first fragmented by using of 2 μL RNA fragmentation buffer (Thermo Fisher, Waltham, MA, USA). 10% of the fragmented RNA was reserved for each sample and set as the input control. Fragmented RNA was incubated with 10 μL m^6^A antibody (3 µg/500 µL, Abcam, No. ab208577) in IP-binding buffer (10 mM Tris-HCl, 150 mM NaCl, 0.1% NP-40, pH 7.4). The mixture was then incubated with 50 μL protein A/G magnetic beads (Thermo Fisher) for 2 h at 4°C. Subsequently, the beads were harvested and washed twice in IP wash buffer (10 mM Tris-HCl, 1 M NaCl, 0.1% NP-40, pH 7.4). The bound RNAs were eluted from the beads with m^6^A elution buffer (0 mM Tris-HCl, 1 M NaCl, 0.1% NP-40, 25 mM m^6^A, pH 7.4). Eluted fragmental RNAs were harvested and purified by an A&DPure Trizol Total RNA Purification Kit (A&D, Ann Arbor, MI, USA). Further enrichment of SNHG1 was calculated by qPCR and the corresponding m^6^A enrichment in each sample was calculated by normalizing the input data.

### RNA immunoprecipitation-qPCR assay

The procedures were performed as previously reported (31) and strictly followed the instructions of a Magna RNA immunoprecipitation (RIP) RNA-Binding Protein Immunoprecipitation kit (Millipore). In short, HOS and U2OS (2 × 10^7^) cells after diverse interventions were collected and then lysed with a RIP lysis buffer (containing 10 μL protease inhibitor and 10 μL RNase inhibitor). In total, 10% of cell lysate was set as the input. Magnetic beads were resuspended and vortexed twice with RIP wash buffer. The magnetic beads were incubated with anti-IgG antibody (1:30, Abcam, No. ab313801), anti-METTL3 antibody (1:50, Abcam, No. ab195352), anti-RBM15 antibody (2 µg/mg of lysate, Abcam, No. ab70549) or anti-EWSR1 antibody (12.03 µg/mL, Abcam, No. ab252829) under rotation for 30 min at room temperature. The labeled magnetic beads were harvested, and 900 μL of RIP immunoprecipitation buffer and 100 μL of cell lysate were added to the labeled magnetic beads and incubated with rotation for 3 h overnight at 4°C. The next day, an RNeasy MinElute Cleanup kit (Qiagen, Valencia, CA, USA) was used to extract the immunoprecipitated RNA, and the extracted RNAs were reversely transcribed and subjected to RT-qPCR to detect the relative abundance of SNHG1.

### RNA pulldown assay

The procedure was executed as previously described (32) by using a Pierce™ Magnetic RNA Protein Pull-Down Kit (Thermo Fisher) according to the manufacturer’s instructions. In brief, the biotin-labeled SNHG1 probe and corresponding vector probe were synthesized by RiboBio Co. Ltd. (Ribobio, Guangzhou, China). HOS and U2OS cells were lysed, and the cell lysates were harvested. Cell lysates were resuspended in pull-down lysis buffer, then homogenized by a homogenizer and centrifuged at 12,000 rpm for 15 min. The supernatants were harvested and incubated with 200 pmol of biotin-labeled RNA probes at 4°C for 4 h, followed by incubation with 20 μL of prepared streptavidin magnetic beads (Thermo Fisher) for another 1 h at 4°C before washing five times with wash buffer (20 mM Tri-HCl, pH 7.5, 1 mM EDTA and 300 mM NaCl). The pulldown proteins were subjected to the following Western blot analysis.

### Chromatin immunoprecipitation-qPCR assay

The procedures were executed as previously reported (33). In brief, 5 × 10^6^ HOS and U2OS cells were cross-linked with 1% formaldehyde for 10 min at room temperature. The cross-linking was ceased by using 0.125 M of glycine after 5 min of incubation at room temperature. The OS cells were rinsed twice with cold PBS (containing 1 mM PMSF and 1 × protease inhibitor cocktail), lysed with a chromatin immunoprecipitation (ChIP) lysis buffer (Cell Signaling Technologies, Danvers, MA, USA), and then sonicated to yield DNA fragments with sizes of 0.2 to 1 kb. In total, 10% of the fragmented DNA was set as the input control. The remaining DNA was incubated overnight at 4°C on a rotator with 10 μg of anti-EWSR1 (Abcam, No. ab252829) or anti-IgG antibody (Abcam, No. ab313801). The next day, ChIP-Grade Protein G Magnetic Beads (Cell Signaling Technologies) were added to the samples and incubated for another 4 h at 4°C. After 4 h, the beads were harvested and washed three times with 1 × ChIP buffer (Cell Signaling Technologies), followed by a single wash with 1 mL lysis buffer containing 1 × ChIP buffer and 0.5 M NaCl using a magnetic separation rack. The chromatin was eluted in a ChIP Elution Buffer (Cell Signaling Technologies) followed by reverse crosslinking at 65°C for at least 4 h. ChIP DNA was treated with 5 g/mL RNase A and 0.2 mg/mL protease K and purified using NucleoSpin Gel and PCR Clean-up (MACHEREY-NAGEL, Düren, Germany). The purified ChIP DNA was quantified by a qPCR assay to detect the abundance of immunoprecipitated WDR74 promoter.

### *In vivo* nude mouse model

The procedure was performed as previously reported (34). Female BALB/c nude mice aged 4–5 weeks were purchased from the Animal Care and Use Committee of Dalian Medical University Ltd. (Dalian, China) and kept under sterile specific-pathogen-free (SPF) conditions. HOS cells (1 ×10^6^, mixed with Matrigel, BD Bioscience, Shanghai, China, 1:1) stably overexpressing METTL3 or the corresponding blank vector were injected subcutaneously or intravenously to construct the *in vivo* tumorigenesis model or *in vivo* lung metastasis model. Formatted subcutaneous tumor nodes were monitored weekly, and lung metastatic nodes were monitored at week four by computed tomography (CT, Siemens, München, Germany). The formatted subcutaneous tumor nodes and metastatic lung nodes were then harvested for further analysis. This study was conducted in accordance with the Guide for the Care and Use of Laboratory Animals of the National Institutes of Health and was approved by the Institute Research Medical Ethics Committee of Shenyang Medical College.

### Statistical analysis

All results are expressed as the mean ± SD from at least three independent experiments. Statistical analysis was performed using GraphPad Prism V6.0 (GraphPad Software Inc., La Jolla, CA, USA). Differences between the two groups were analyzed using an unpaired two-tailed Student’s *t*-test. Differences among multiple groups were assessed using one-way ANOVA, and when significance was observed, the criterion for significance was set as *p* < 0.05. ^*^ *p* < 0.05, ^**^ *p* < 0.01, ^***^ *p* < 0.001; ^****^ *p* < 0.0001, ^#^ *p* < 0.05, ^##^ *p* < 0.01, ^###^ *p* < 0.001, and ^####^ *p* < 0.0001, respectively.

## Results

### METTL3 is upregulated in OS

**Figure 1:**
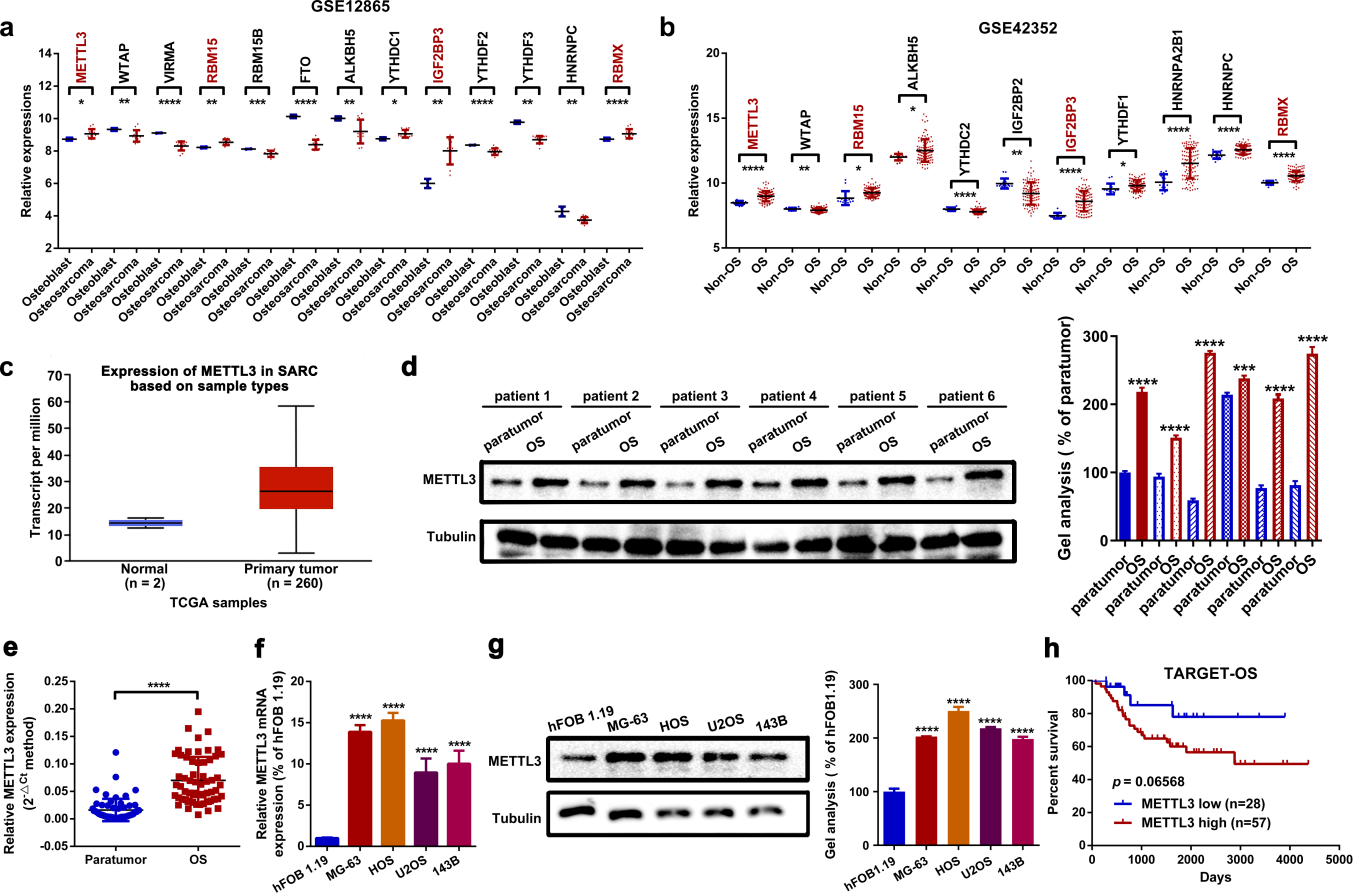
METTL3 is upregulated in OS. **(a, b)** Dysregulated m^6^A-related genes in two OS-related GEO datasets (GSE12865 and GSE42352) were analyzed using GEO2R. METTL3, RBM15, IGF2BP3, and RBMX were found to be upregulated in OS (^*^ *p* < 0.05; ^**^ *p* < 0.01; ^***^ *p* < 0.001; ^****^ *p* < 0.0001, respectively). **(c)** METTL3 was upregulated in 260 sarcoma samples compared to two normal tissue samples, based on online TCGA analysis by using the web tool UALCAN. **(d, e)** Expression of METTL3 in OS tissue was measured by Western blot **(d)** and RT-qPCR assay **(e)**. ^****^ *P* < 0.0001. **(f, g)** Expression of METTL3 in the normal osteoblast cell line hFOB1.19 and in four OS cell lines (MG-63, HOS, U2OS, and 143B) was determined by RT-qPCR **(f)** and Western blot **(g)** (^****^ *p* < 0.0001). **(h)** The overall survival rate of OS patients with diverse METTL3 expression levels was analyzed by Kaplan–Meier survival analysis according to an online analysis of TCGA database (TARGET-OS) by using an online web tool PCAT. All data are presented as mean ± SD from three independent experiments.

We initially focused on the dysregulated m^6^A-related genes through an online analysis of OS-related GEO datasets GSE12865 and GSE42352. As shown in **Figures 1a, b**, several m^6^A genes, including METTL3, RBM15, IGF2BP3, and RNA-binding motif protein X (RBMX), were significantly upregulated in OS cell lines and OS tissues compared to osteoblast and non-osteosarcoma tissues. We primarily concentrated on the role of METTL3, a well-known m^6^A “writer”, in OS. Based on an online analysis of TCGA database, we found that METTL3 was upregulated in sarcoma tissue samples (*n* = 260) compared to normal tissues (*n* = 2) (**Figure 1c**). Clinically, our own analysis using Western blot and RT-qPCR assays demonstrated that METTL3 was significantly upregulated in OS tissues (**Figures 1d, e**). Furthermore, METTL3 was also upregulated in four OS cell lines, with hFOB 1.19 used as a control (**Figures 1f, g**). Through an online analysis of TCGA database (TARGET-OS) using the web tool PCAT (35), it was found that the overall survival rate of patients with high METTL3 expression (*n* = 57) was lower than that of patients with low METTL3 expression (*n* = 28) (**Figure 1h**).

### METTL3 functioned as an oncogenic regulator of proliferation and migration in OS cells by enhancing SNHG1 stability *in vitro*

**Figure 2:**
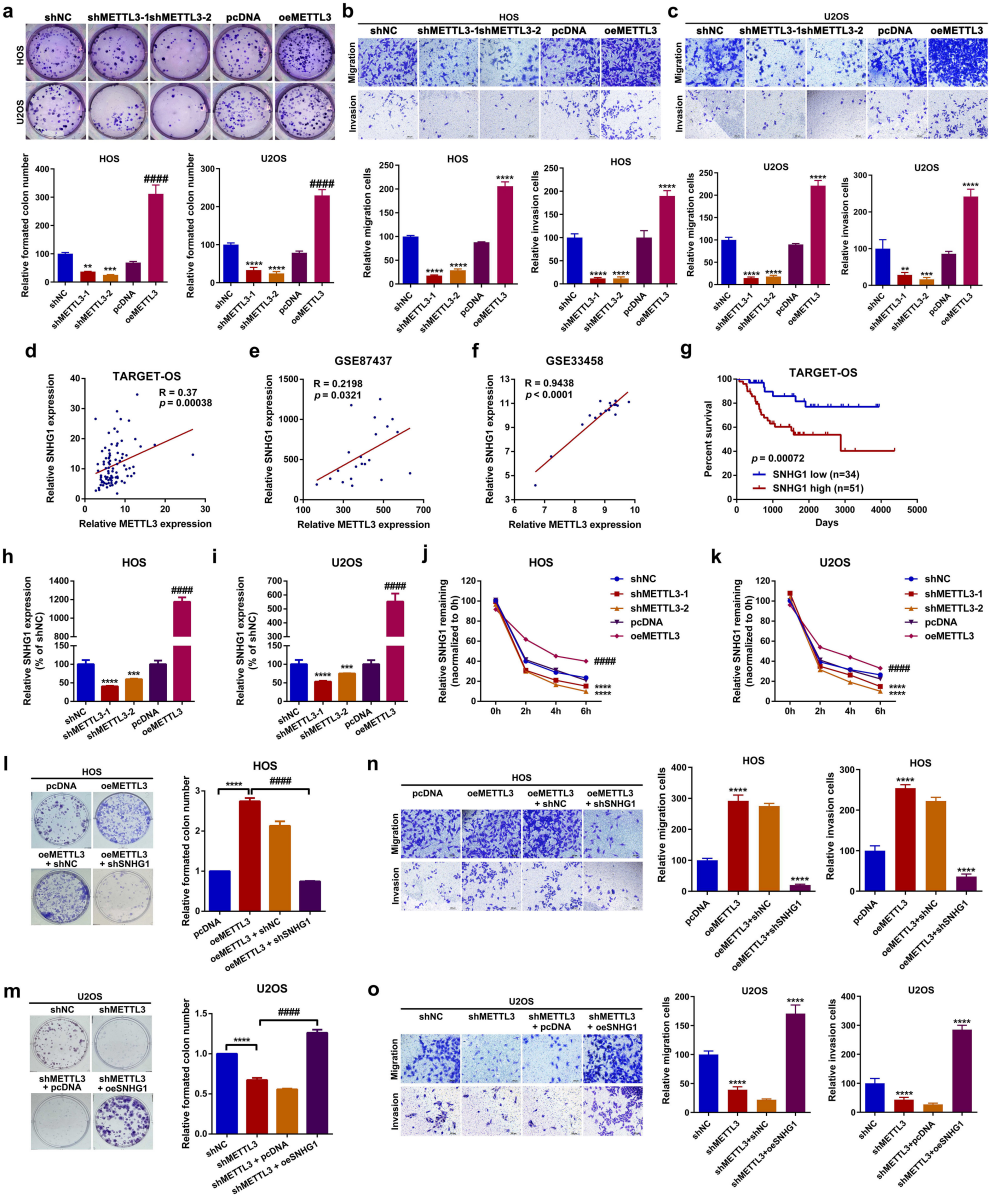
METTL3 functioned as an oncogenic regulator of proliferation and migration in OS by enhancing the stability of SNHG1 *in vitro*. **(a)** Changes in cell proliferation ability in HOS and U2OS cells after up- and downregulation of SNHG1 were analyzed by colony formation assay. **P < 0.01,***P < 0.001,****P < 0.0001 and ^####^ *P* < 0.0001 as compared with shNC and pcDNA, respectively. **(b**, **c)** Changes in cellular motility in HOS and U2OS cells following up- and downregulation of SNHG1 were evaluated by transwell assay. **P < 0.01,***P < 0.001 and ****P < 0.0001 as compared with shNC and pcDNA, individually. Magnification, × 400; scale bar, 200 μm. **(d–f)** The correlation between METTL3 and SNHG1 in TARGET-OS, GSE87437, and GSE33458 was analyzed by Spearman correlation analysis. **(g)** Kaplan–Meier overall survival curves for SNHG1 of human OS, *p* = 0.00072 as analyzed by a log-rank test. **(h**, **i)** SNHG1 expression after various METTL3 interventions was assessed by RT-qPCR assay. ^***^ *p* < 0.001 and ^****^ *p* < 0.0001 as compared with shNC; ^####^ *p* < 0.0001 as compared with pcDNA, respectively. **(j**, **k)** SNHG1 stability following different METTL3 interventions was checked by an actinomycin D assay. ^****^ *p* < 0.0001 and ^####^ *p* < 0.0001 as compared with shNC or pcDNA, separately. **(l**, **m)** Colony formation assays were conducted to evaluate changes in cell proliferation in HOS and U2OS cells. ****P < 0.0001. **(n**, **o)** Transwell assays were performed to assess cell motility changes in HOS and U2OS cells. ^**^ *p* < 0.01 as compared with pcDNA or shNC, and ^##^ *p* < 0.01 as compared with oeMETTL3 and shMETTL3, respectively. Magnification, × 400; scale bar, 200 μm. All data are presented as the mean ± SD from three independent experiments.

In this section, we explored the role of METTL3 in OS cell proliferation and migration. Functionally, it was demonstrated that both up- and downregulation of METTL3 promoted or suppressed the proliferation and migration abilities of HOS and U2OS cells, respectively (**Figures 2a–c**, **Supplementary Figures S1a–d**). These findings suggest that METTL3 functions as an oncogenic regulator in OS cell proliferation and migration. As a well-known m^6^A “writer”, METTL3-mediated m^6^A modification is closely correlated with RNA metabolism, including lncRNAs, in cancer (36). Through online analysis of TCGA database (TARGET-OS) using PCAT and reanalysis of two OS-related GEO datasets, GSE87437 and GSE33458, we examined the relationship between METTL3 and several lncRNAs implicated in OS, including DANCR, taurine upregulated 1 (TUG1), SNHG1, urothelial cancer-associated 1 (UCA1), breast cancer antiestrogen resistance 4 (BCAR4), small nucleolar RNA host gene 5 (SNHG5), tumor suppressor candidate 7 (TUSC7), MALAT1, maternally expressed 3 (MEG3), and growth arrest-specific 5 (GAS5), using Spearman correlation analysis. As shown in **Supplementary Table S2** and **Figures 2d–f**, SNHG1 was selected for further study due to its positive correlation with METTL3 across all three databases. Meanwhile, we found that patients with high SNHG1 expression had significantly shorter overall survival rates compared to those with low SNHG1 expression (**Figure 2g**). As previously reported, the oncogenic role of SNHG1 in OS has been well-explored (15, 17–19). Based on these findings, we hypothesized that METTL3 may influence OS cell proliferation and migration through the regulation of SNHG1. A further RT-qPCR assay indicated that up- and downregulation of METTL3 affected the expression levels of SNHG1 (**Figures 2h, i**) in OS cells. METTL3 is reported to regulate RNA stability in an m^6^A-dependent manner (10, 37, 38). To further investigate this, an actinomycin D assay was conducted to assess the effect of METTL3 on SNHG1 stability. As shown in **Figures 2j, k**, the knockdown of METTL3 significantly accelerated SNHG1 decay. Conversely, the upregulation of METTL3 enhanced the stability of SNHG1. Lastly, through SNHG1-related functional assays, we observed that upregulation of METTL3 (oeMETTL3) promoted proliferation and migration in OS cells. This facilitative effect of oeMETTL3 was attenuated by knockdown of SNHG1 (oeMETTL3 + shSNHG1) (**Figures 2l, n**; **Supplementary Figure S1E**). Similarly, we demonstrated that downregulation of METTL3 (shMETTL3) suppressed proliferation and migration in OS cells, and this suppressive effect was reversed by upregulation of SNHG1 (shMETTL3 + oeSNHG1) (**Figures 2m, o**; **Supplementary Figure S1F**). Taken together, these data indicate that SNHG1 is, at least partially, a downstream target in METTL3-mediated proliferation and migration in OS cells.

### METTL3 catalyzes m^6^A modification of SNHG1 with the assistance of RBM15

**Figure 3:**
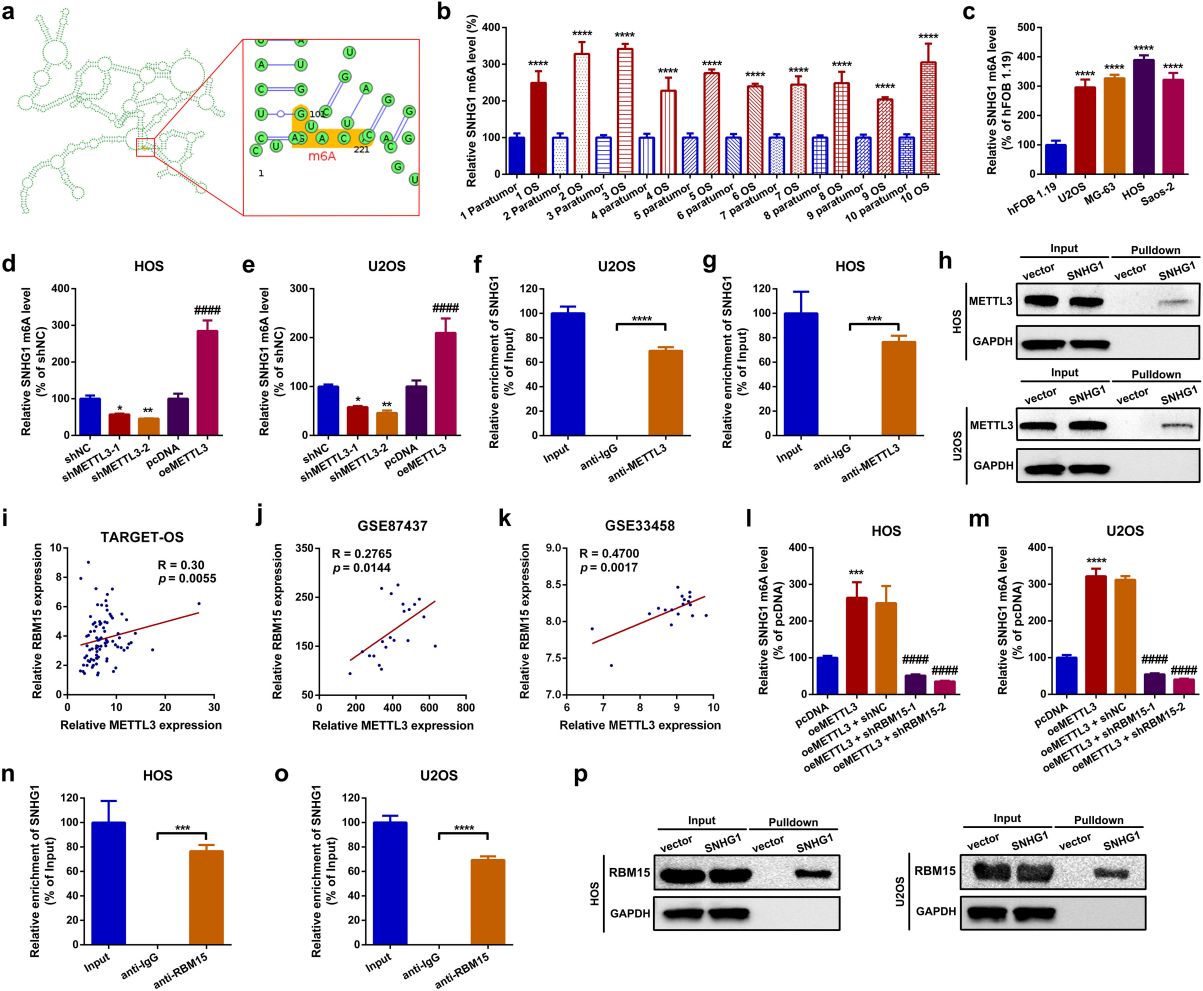
METTL3 catalyzed m^6^A modification of SNHG1 with the assistance of RBM15. **(a)** Predicted m^6^A sites of SNHG1 identified using the online web tool SRAMP. **(b)** The m^6^A level of SNHG1 in 10 OS tissue specimens and their paired paratumor tissue specimens was qualified by a MeRIP-qPCR assay. ^****^ *p* < 0.0001 as compared with paratumor. **(c)** The m^6^A level of SNHG1 in the normal osteoblast cell line hFOB1.19 and in four OS cell lines was determined by MeRIP-qPCR. ^****^ *p* < 0.0001 as compared with hFOB1.19. **(d, e)** The m^6^A level of SNHG1 in HOS and U2OS after different METTL3 interventions was assessed by MeRIP-qPCR. ^*^ *p* < 0.05 and ^**^ *p* < 0.01 as compared with shNC; ^####^ *p* < 0.0001 as compared with pcDNA. **(f, g)** An RIP assay was performed to verify the targeted binding effect between SNHG1 and METTL3 in HOS and U2OS cells. ^***^ *p* < 0.001 and ^****^ *p* < 0.0001 as compared with anti-IgG. **(h)** An RNA pulldown assay was conducted to confirm the binding interaction between SNHG1 and METTL3 in HOS and U2OS cells. **(i–k)** The correlation between METTL3 and RBM15 in TARGET-OS, GSE87437, and GSE33458 datasets was analyzed using Spearman correlation analysis. **(l**, **m)** The m^6^A level of SNHG1 in HOS and U2OS after different METTL3 and RBM15 interventions was measured by MeRIP-qPCR. ^****^ *p* < 0.0001 and ^####^ *p* < 0.0001 as compared with pcDNA or oeMETTL3, respectively. **(n**, **o)** The binding interaction between SNHG1 and RBM15 in HOS and U2OS cells was verified by RIP assay. ^***^ *p* < 0.001 and ^****^ *p* < 0.0001 as compared with anti-IgG. **(p)** Another RNA pulldown assay was used to confirm the binding effect between SNHG1 and RBM15 in HOS and U2OS cells. All data are presented as the mean ± SD from three independent experiments.

In this section, using the online m^6^A modification prediction web tool SRAMP (39), we first identified that SNHG1 contains an “RRACH” motif at positions 101–105 (**Figure 3a**; **Supplementary Figure S2a**). Meanwhile, through a methylated RNA immunoprecipitation qPCR (MeRIP-qPCR) assay, we demonstrated that the m^6^A modification of SNHG1 was abundantly enriched in OS tissues and cell lines (**Figures 3b, c**). Furthermore, we found that up- and downregulation of METTL3 positively affected the m^6^A level of SNHG1 in OS cells (**Figures 3d, e**). Next, an RIP assay was performed to confirm the interaction between METTL3 and SNHG1. As shown in **Figures 3f, g**, compared with IgG, the enrichment of SNHG1 was significantly higher with the METTL3 antibody. In addition, an RNA pull-down assay was conducted to verify the direct binding between METTL3 and SNHG1. As shown in the representative images in **Figure 3h**, using GAPDH as a control, METTL3 was pulled down and then detected in the biotin-labeled SNHG1 group but not in the vector group. Previous studies have reported that RBM15 can recruit MTCs to certain lncRNAs, such as X inactive specific transcript (XIST), thereby assisting their anchoring to MTCs (40). Encouragingly, through an online analysis of TCGA database (TARGET-OS) and a reanalysis of two GEO datasets, GSE87437 andGSE33458, we found that RBM15 expression was positively correlated with METTL3 expression in OS (**Figures 3i–k**). To confirm whether RBM15 is involved in METTL3-mediated SNHG1 m^6^A modification, we knocked down RBM15 in METTL3-overexpression OS cells (**Supplementary Figures S2b–d**), and the m^6^A-modified SNHG1 was then analyzed using a MeRIP-qPCR assay. As we speculated, the knocked down of RBM15 reduced the m^6^A level of SNHG1 (**Figures 3l, m**). Additionally, using an RIP assay and an RNA pull-down assay, we found that SNHG1 interacted with RBM15 through direct binding (**Figures 3n–p**). These findings indicate that RBM15 assists METTL3 in catalyzing the m^6^A modification of SNHG1.

### SNHG1 promotes proliferation and migration by regulating its neighboring gene, WDR74, in OS cells

**Figure 4:**
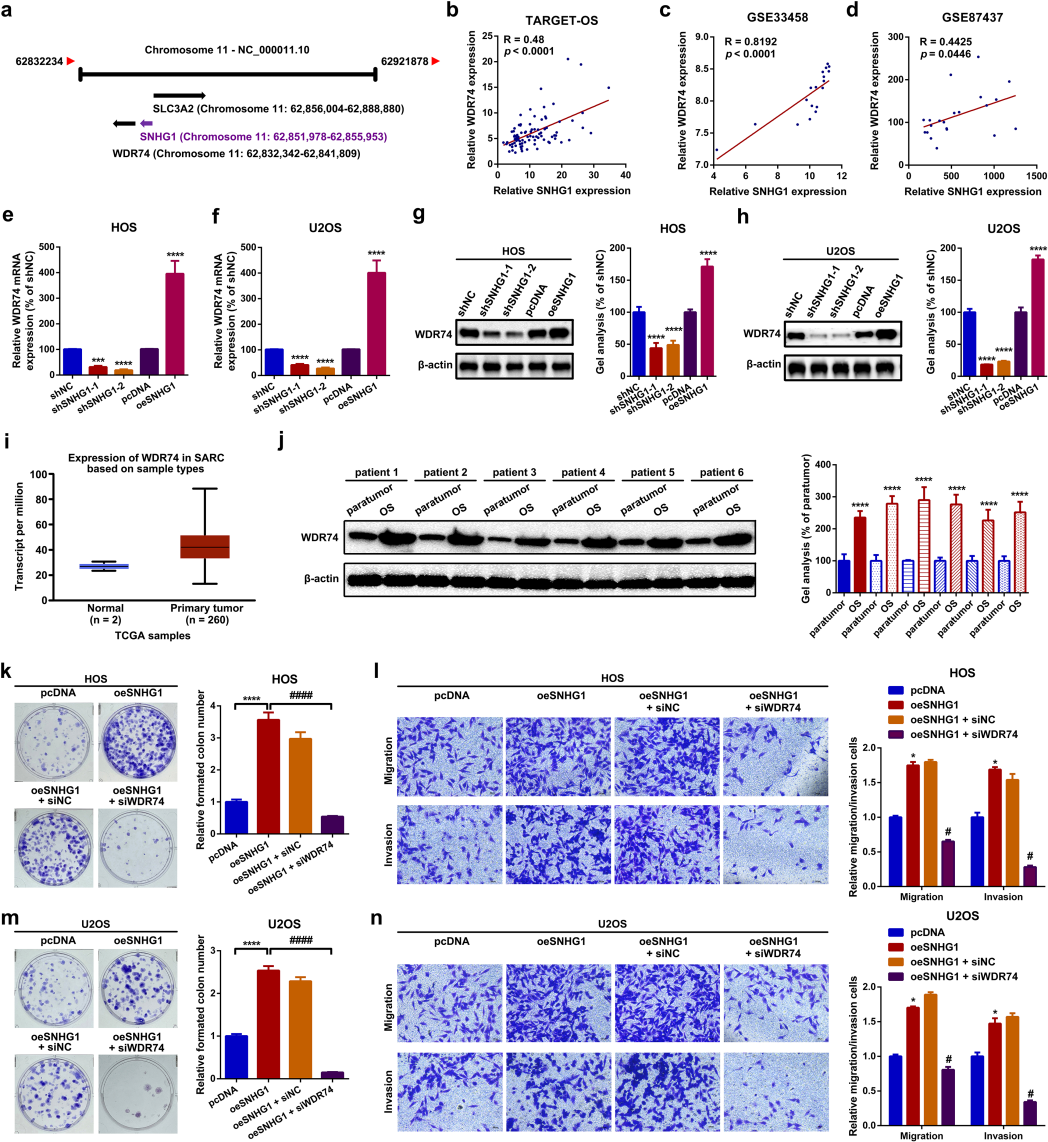
SNHG1 promoted proliferation and migration via regulation of its neighboring gene WDR74 in OS cells. **(a)** A diagram showing the neighboring genes of SNHG1. **(b–d)** Spearman correlation analyses revealed a positive correlation between SNHG1 and WDR74 in the TARGET-OS, GSE33458, and GSE87437 datasets. **(e–h)** The mRNA and protein expression levels of WDR74 following different SNHG1 interventions were measured by RT-qPCR. ***P < 0.001 and ****P < 0.0001 as compared with shNC and pcDNA. **(i)** The expression of WDR74 in 260 sarcoma samples and two normal tissue samples was analyzed using the UALCAN web tool. **(j)** WDR74 expression in six OS tissue specimens and paired para tumor tissue specimens was qualified by a Western blot assay. ^****^ *p* < 0.0001 as compared with paratumor. **(k**, **m)** Changes in cell proliferation in HOS and U2OS cells were evaluated by a colony formation assay. ^****^ *p* < 0.0001 and ^####^ *p* < 0.0001 as compared with pcDNA or oeSNHG1, respectively. **(l**, **n)** Cell motility changes in HOS and U2OS cells were analyzed by a transwell assay. ^*^ *p* < 0.05 and ^#^ *p* < 0.05 as compared with pcDNA or oeSNHG1, respectively. Magnification, × 400; scale bar, 200 μm. All data are presented as the mean ± SD from three independent experiments.

In this section, by a localization FISH assay, we found that SNHG1 was mainly located at the nucleus but not at the cytoplasm (**Supplementary Figure S3a**). This phenomenon indicated that SNHG1 might serve as a transcriptional regulator in OS cells. Recent research has demonstrated that lncRNAs are involved in cancerous disease via regulating its neighboring genes (41, 42). We here attempted to explore whether SNHG1 might regulate SLC3A2 and WDR74, two neighboring genes of SNHG1 (**Figure 4a**; **Supplementary Figure S3b**). Firstly, we uncovered that the expression of SNHG1 was closely correlated with WDR74 but not with SLC3A2 via online correlation analysis (**Figures 4b–d**; **Supplementary Figures S3c–e**). Therefore, WDR74 was selected in the following research. Functionally, it was displayed that up- and downregulation of SNHG1 correspondingly increased and decreased WDR74 expression both at the mRNA and at the protein level (**Figures 4e–h**). Next, we found that WDR74 was upregulated in OS (**Figure 4i, j**). Functionally, we found that the knockdown of WDR74 attenuated the facilitative effect on proliferation and migration mediated by SNHG1 (**Figures 4k–n**). In short, the uncovering of this section suggested that SNHG1 promoted proliferation and migration via regulation of its neighboring gene WDR74 in OS cells.

### SNHG1 affected WDR74 transcription through EWSR1 recruitment

**Figure 5:**
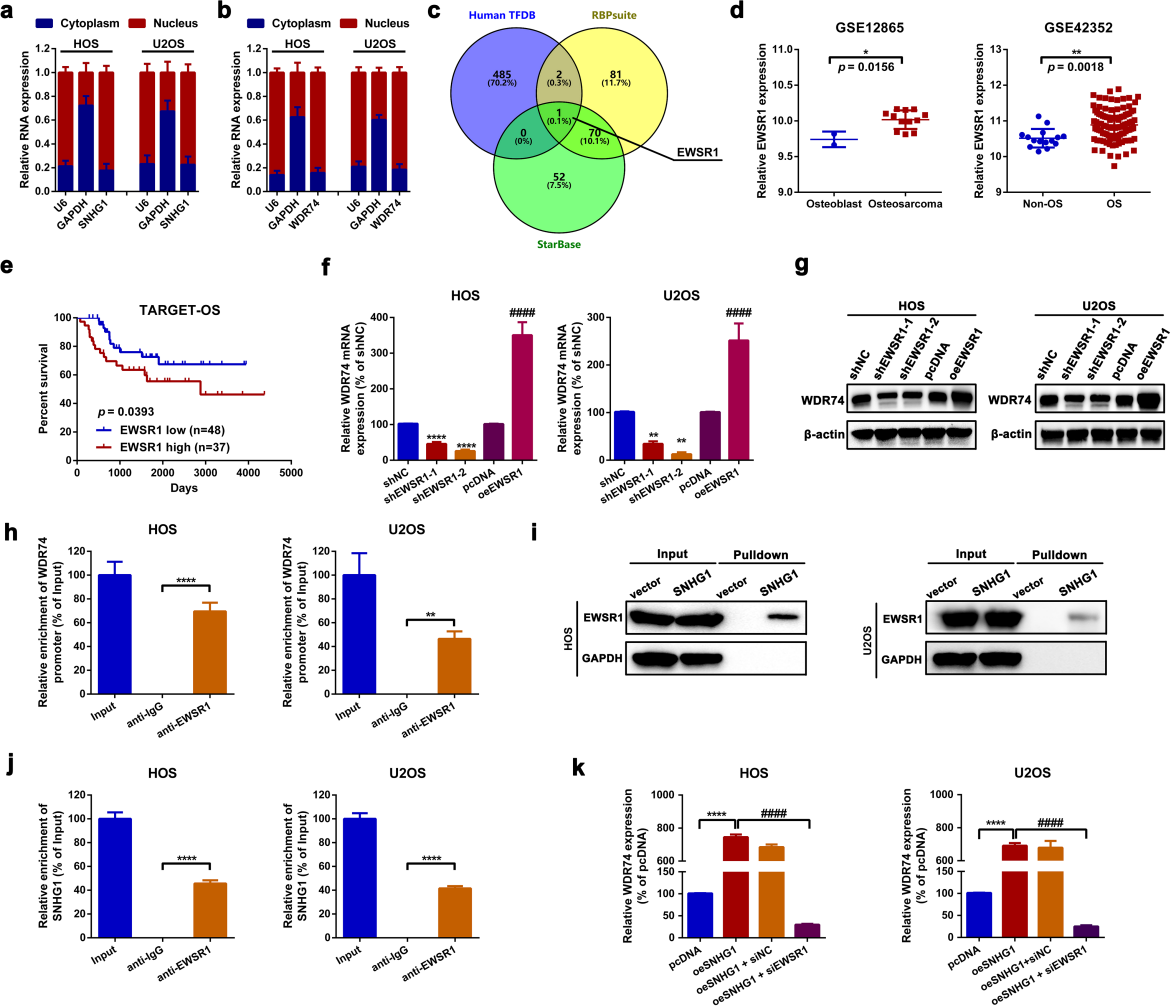
SNHG1 affected WDR74 transcription by EWSR1 recruitment. **(a, b)** Cytosolic/nuclear fractionation assays were conducted to determine the subcellular localization of SNHG1 and WDR74. **(c)** RBPs potentially interact with SNHG1 and WDR74 promoters were predicted using human TFBD, StarBase, and RBPsuite. **(d)** EWSR1 expression in two OS-related GEO datasets (GSE12865 and GSE42352) was analyzed using GEO2R. ^*^ *p* < 0.05 and ^**^ *p* < 0.01 as compared with osteoblast or non-OS, respectively. **(e)** Kaplan–Meier analysis was used to assess the overall survival rate in patients with different EWSR1 expression levels as calculated by a log-rank test. **(f, g)** WDR74 mRNA and protein expression following various EWSR1 interventions were measured by an RT-PCR assay and a western blot assay, respectively. ^****^ *p* < 0.0001 and ^####^ *p* < 0.0001 as compared with shNC or pcDNA, respectively. **(h)** A ChIP-qPCR assay was performed to evaluate the binding of EWSR1 to the WDR74 promoter. ^****^ *p* < 0.0001 as compared with anti-IgG. **(i, j)** RNA pulldown and RIP assays were used to verify the binding effect between SNHG1 and EWSR1. ^****^ *p* < 0.0001 as compared with anti-IgG. **(k)** WDR74 mRNA expression following various SNHG1 and EWSR1 interventions was assessed by RT-qPCR. ^****^ *p* < 0.0001 and ^####^ *p* < 0.0001 as compared with oeSNHG1. All data are presented as the mean ± SD from three independent experiments.

Through a cytosolic/nuclear fractionation assay by using GAPDH mRNA as cytoplasmic control and U6 RNA as nuclear control, it was unveiled that both SNHG1 and WDR74 were mainly expressed in the nucleus rather than in the cytoplasm (**Figures 5a, b**) in HOS and U2OS cells. Combined with the findings presented above in **Figures 4g–j**, it was indicated that SNHG1 impacted WDR74 expression on the transcriptional level. LncRNAs are reported to regulate transcription via the recruitment of regulatory protein complexes. By using human TFDB (http://bioinfo.life.hust.edu.cn/HumanTFDB#!), RBP suites (http://www.csbio.sjtu.edu.cn/bioinf/RBPsuite/) and Starbase, EWS RNA-binding protein 1 (EWSR1) was selected as the only RBP that might interact with SNHG1 and the promoter region of WDR74 (NC_000011.10:c62843809-62841809, **Supplementary Table S3**) (**Figure 5c**). EWSR1, also named EWS-FLI1, is well-reported as a transcription factor in Ewing sarcoma (43). Here, according to an online bioinformatics analysis of GSE12865, GSE42352, and TARGET-OS, we unveiled that EWSR1 was upregulated and that high expression of EWSR1 was correlated with shorter survival rates in patients with OS (**Figures 5d, e**). Functionally, we found that up- and downregulation of EWSR1 significantly increased or decreased the expression of WDR74 (**Figures 5f, g**) in OS cells. Furthermore, a ChIP-qPCR assay was applied to confirm the binding effect between EWSR1 and the promoter region of WDR74. As the data shown in **Figure 5h**, the promoter region of WDR74 was remarkably enriched in the EWSR1 antibody but not in the IgG antibody. Moreover, an RNA pull-down assay and an RIP assay were performed to examine the potential interaction between SNHG1 and EWSR1. As shown in the representative images in **Figure 5i**, a greater amount of EWSR1 protein was pulled down by the biotin-labeled SNHG1 probe. Also, it was found that SNHG1 was significantly enriched in an anti-EWSR1 group rather than in an anti-IgG group (**Figure 5j**). Lastly, it was demonstrated that the knockdown of EWSR1 remarkably attenuated the promotive effect of SNHG1 on WDR74 transcription (**Figure 5k**). Taken together, the findings of the current section indicated that SNHG1 affected WDR74 transcription through EWSR1 recruitment.

### METTL3 promotes OS tumorigenesis and lung metastasis *in vivo*

**Figure 6:**
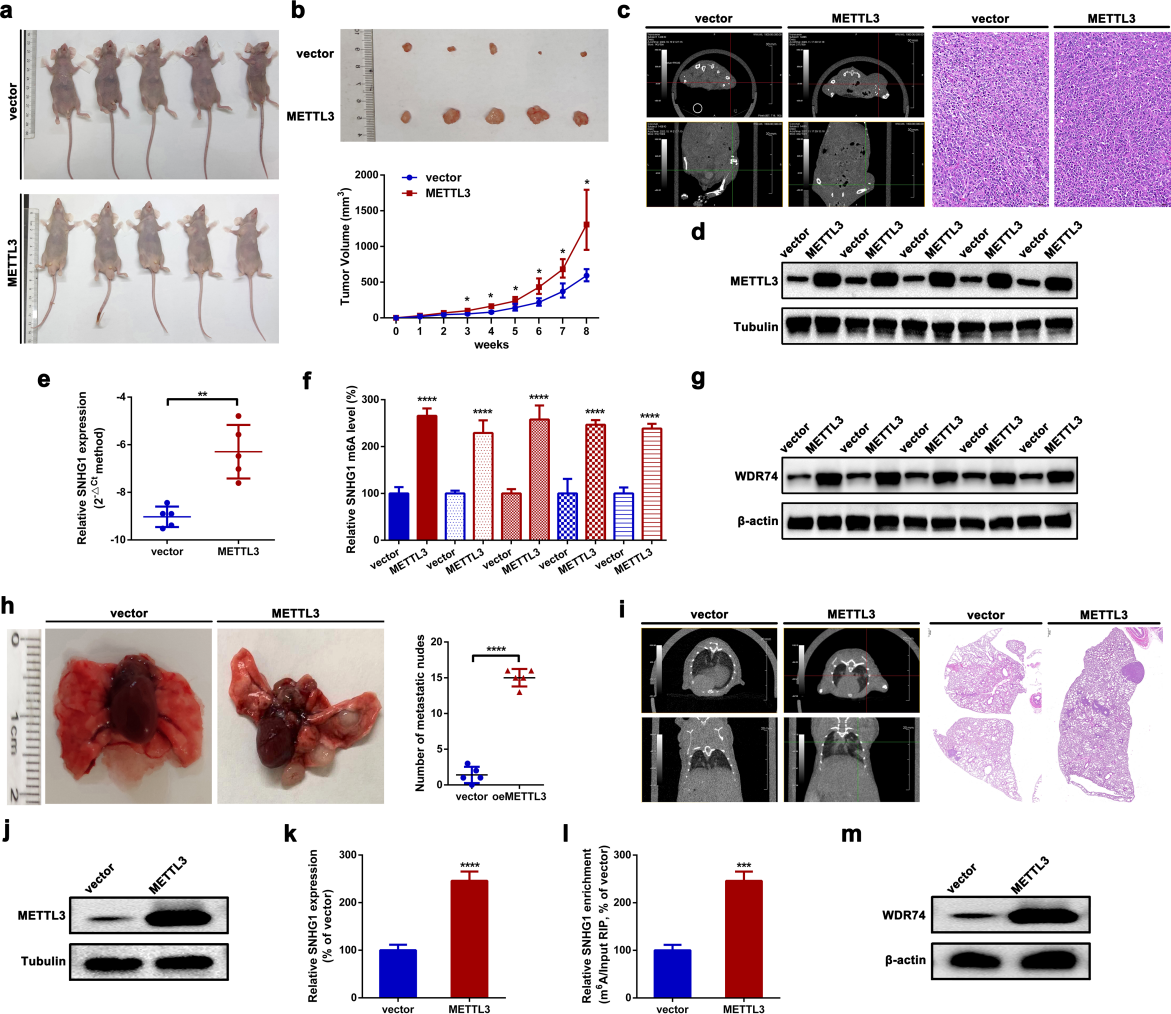
METTL3 promotes OS tumorigenesis and lung metastasis *in vivo*. **(a)** Macroscopic view of nude mice subcutaneously injected with HOS cells stably overexpressing METTL3, with HOS cells carrying an empty vector used as a control. **(b)** Representative images of resected xenograft tumors from nude mice with different METTL3 interventions (top); growth curves of subcutaneous tumors based on weekly tumor volume measurements (bottom). ^*^ *p* < 0.05 as compared with vector. **(c)** Representative CT scan (left) and H&E staining (right; scale bars, 200 µm; magnification, × 100) of subcutaneous tumors in nude mice following different METTL3 interventions. Magnification, × 20; scale bar, 50 μm. **(d–g)** The expression of METTL3 and SNHG1, the m^6^A modification level of SNHG1, and the expression of WDR74 in xenograft tumors were assessed using Western blot, an RT-qPCR, MeRIP-qPCR, and Western blot assays, respectively. ^**^ *p* < 0.01 and ^****^ *p* < 0.0001 as compared with vector. **(h)** Representative images of lung metastatic nodes in nude mice after different METTL3 interventions (left); quantification of metastatic nodes (right). ^****^ *p* < 0.0001 as compared with the vector group. **(i)** Representative CT scan (left) and H&E staining (right; scale bar, 500 µm; magnification, × 10) of lung metastatic in nude mice following different METTL3 interventions. Magnification, × 2; scale bar, 500 μm. **(j–m)** The expression levels of METTL3 and SNHG1, the m^6^A modification level of SNHG1, and the expression of WDR74 in lung metastatic nodes were measured by Western blot, RT-qPCR, MeRIP-qPCR, and Western blot assays, respectively. ***P < 0.001 and ****P < 0.0001 as compared with vector. All data are presented as the mean ± SD from three independent experiments.

In this section, orthotopic xenograft mouse models were constructed to evaluate the role of METTL3 in OS tumorigenesis and lung metastasis *in vivo*. As shown in **Figures 6a, b**, the upregulation of METTL3 significantly facilitated OS tumor growth in nude mice. Meanwhile, tumor growth was assessed using an animal CT scan. As depicted in **Figure 6c**, overexpression of METTL3 notably promoted OS tumor growth in mice. Meanwhile, the expression levels of METTL3, SNHG1, and WDR74 in the formatted subcutaneous nudes were detected by an RT-qPCR assay and a Western blot assay, respectively. As shown in **Figures 6d–g**, the expression of METTL3, SNHG1, and WDR74 were significantly higher in the formatted nudes from METTL3-overexpressing xenografts compared to those from vector xenografts. Even more, the pulmonary metastasis model of OS in mice was also constructed. As the typical photographs displayed in **Figure 6h**, upregulation of METTL3 promoted metastatic nude formation in the lungs. Meanwhile, an animal CT scan clearly showed that upregulation of METTL3 promoted OS lung metastasis in mice (**Figure 6i**). Finally, it was found that the expression of METTL3, SNHG1, and WDR74 in the formatted metastatic nudes in METTL3 xenografts was remarkably higher than that in vector xenografts (**Figures 6j–m**). Together, all the findings above suggested that overexpression of METTL3 promoted SNHG1 and WDR74 expression, and upregulation of METTL3 facilitated OS tumorigenesis and lung metastasis *in vivo*.

## Discussion

METTL3, a key methyltransferase subunit, is primarily composed of a zinc finger domain (ZFD) and a methyltransferase domain (MTD) (44). METTL3 and its partner METTL14 mainly constitute the core component of MTCs, which participate in aspects of RNA metabolism like alternative splicing, transport, stability, microRNA maturation, and decay (5, 45). METTL3-initiated m^6^A presents contradictory roles in RNA stability depending on diverse readers (46, 47). In the present study, we found that METTL3 enhances the stability of SNHG1 in an m^6^A-dependent manner, suggesting the involvement of m^6^A reader proteins in this process. Among these readers, IGF2BP3 has been reported to recognize m^6^A-modified transcripts and promote their stability, although its role in RNA stabilization remains somewhat controversial (47). Notably, as shown in **Figures 1a, b**, IGF2BP3 is consistently upregulated in OS tissues. Based on these observations, we hypothesize that IGF2BP3 may act as an m6A reader that protects SNHG1 from degradation. Moreover, its overexpression in OS may account for the increased SNHG1 stability observed in our experimental model. The functional role of METTL3 in cancers is debatable (5, 48, 49). Similar to several previous reports (12, 50–52), the current research showed that METTL3 acts as an oncogenic regulator in OS cell proliferation and migration. Among the components of MTCs, METTL3 is the only catalytic subunit via its special S-adenosylmethionine (SAM) as the methyl donor (44, 53). The findings of the present study also demonstrated that METTL3 affected the m^6^A level of SNHG1.

Not only is the METTL3-METTL14 heterodimer part of the complex, but MTCs also include other binding partners such as WT1-associated protein (WTAP), zinc finger CCCH-type containing 13 (ZC3H13), vir-like m^6^A methyltransferase associated (VIRMA), and RBM15/15B (40, 54–56). RBM15, acting as a “writer”, is responsible for recruiting MTCs to specific lncRNAs like XIST, thereby promoting XIST methylation (40). In the present study, using RNA pulldown and RIP assays, we first identified that RBM15 can directly bind to SNHG1. Meanwhile, we demonstrated that knockdown of RBM15 reduced the METTL3-mediated methylation of SNHG1. Our findings are the first to illustrate the role of RBM15 working in METTL3-initiated m^6^A modification of SNHG1.

Accumulating evidence has strongly uncovered the oncogenic role of SNHG1 in malignancies (13, 14). As a well-known oncogene, the mechanism of how SNHG1 works is mainly focused on ceRNA, a theory first proposed by Leonardo Salmena (57). SNHG1 is reported to bind to certain RBPs like heterogeneous nuclear ribonucleoprotein L (HNRNPL) and matrin 3 (MATR3) and to promote the progression of prostate cancer (PCa) as well as neuroblastoma (58, 59). SNHG1 also functions as a transcriptional regulator to promote the transcription of its neighboring gene SLC3A2 in *cis* in gastric cancer, colorectal cancer, liver cancer, and lung cancers (25). In the current research, we also focused on the regulatory role of SNHG1 in its neighboring genes in OS. We primarily showed that SNHG1 transcriptionally impacted WDR74 expression in OS. WDR74 is implicated in tumorigenesis, especially in tumor growth and metastasis (60–63). The present research first unveiled the oncogenic role of WDR74 working in OS. We showed that WDR74, acting as a downstream target of SNHG1, was closely involved in SNHG1-mediated proliferation and migration in OS cells. Furthermore, we demonstrated that SNHG1 regulated WDR74 transcription through the recruitment of EWSR1.

As a well-known multifunctional RBP, EWSR1 closely plays a key role in RNA metabolism through its interaction with RNA polymerase II and its coupling with the splicing machinery (64). EWSR1, which belongs to the TET family, participates in various cellular processes by epigenetically regulating gene expression, RNA processing, and cellular signal transduction (65). The crucial role of EWSR1 in Ewing sarcoma has been extensively explored. In the present study, we focus on the expression and function of EWSR1 in OS. We showed that EWSR1 was upregulated in OS. More deeply, we first displayed that EWSR1, acting as a transcription factor, regulated WDR74 expression via binding to the promoter region of WDR74. It is well-accepted that EWSR1 can recruit RNA polymerase II and promote RNA transcription (64, 66, 67). It can be inferred that the promotion of WDR74 transcription was associated with EWSR1-mediated recruitment of RNA polymerase II.

**Figure 7:**
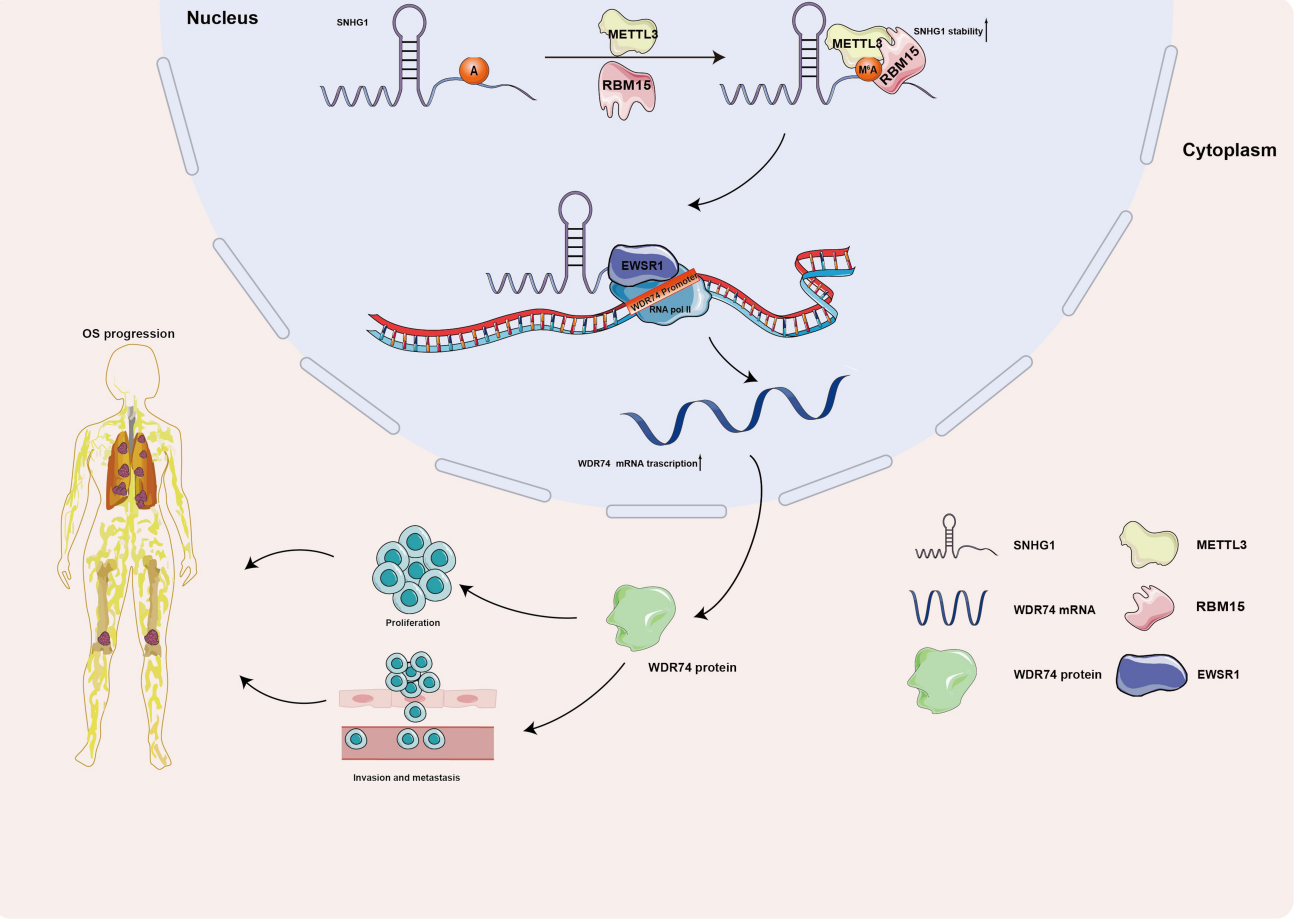
A schematic diagram illustrating the role of the METTL3/SNHG1/WDR74 axis in OS. METTL3 enhances the stability of SNHG1 in an m^6^A-dependent manner with the assistance of RBM15. SNHG1 regulates its neighboring gene, WDR74, in *cis* through the recruitment of EWSR1, ultimately promoting WDR74-mediated proliferation and migration in OS.

Taking all, the current research systematically explored a novel mechanism of how METTL3 works in OS. The present research illustrated that METTL3 enhanced the stability of SNHG1 with the assistance of RBM15 in an m^6^A-dependent manner. And SNHG1, promoted the transcription of WDR74 in *cis*, via recruitment of EWSR1, thereby facilitating WDR74-mediated proliferation and migration (**Figure 7**).

Compared to previous studies, our work presents several key innovations: (1) RBM15 is identified as a specific adaptor mediating METTL3-dependent m^6^A deposition on SNHG1, differing from the classical METTL3–METTL14/WTAP complex (40). (2) SNHG1 activates WDR74 transcriptionally through EWSR1 recruitment, which contrasts with its commonly known ceRNA role in other cancers (19, 25). (3) Most importantly, this is the first report to identify WDR74 as an oncogene in osteosarcoma, suggesting new therapeutic targets. These findings highlight the plasticity of the m^6^A–lncRNA regulatory network in tumor biology and the context-dependent roles of METTL3 and SNHG1 (25, 54). In the future, we will further explore whether IGF2BP3 functions as a downstream effector that mediates the m^6^A-dependent stabilization of SNHG1 in osteosarcoma. Planned experiments include: (1) co-immunoprecipitation (Co-IP) to identify interactions among METTL3, RBM15, and IGF2BP3; (2) m^6^A-RIP-qPCR to test whether IGF2BP3 preferentially binds to m^6^A-modified SNHG1; and (3) CRISPR-based deletion of IGF2BP3 RNA-binding domains to verify its regulatory function.

## Data Availability

The original contributions presented in the study are included in the article/**Supplementary Material**. Further inquiries can be directed to the corresponding authors.

## Supplementary material

Supplementary Figure 1
**(a, c)** Expression of METTL3 mRNA after transfection of specific shRNAs targeted METTL3 or stable overexpression plasmids carrying METTL3 in HOS and U2OS cells was detected by an RT-qPCR assay. ^****^ *P* < 0.0001 and ^****^ *P* < 0.0001 as compared with shNC or pcDNA, individually. **(b, d)** Expression of METTL3 protein after transfection of specific shRNAs targeted METTL3 or stable overexpression plasmids carrying METTL3 in HOS or U2OS cells was detected by an RT-qPCR assay. ^****^ *P* < 0.0001 and ^****^ *P* < 0.0001 as compared with shNC or pcDNA, separately. **(e, f)** Expression of SNHG1 after transfection of specific shRNAs targeted SNHG1 or stable overexpression plasmids carrying SNHG1 in HOS or U2OS cells was detected by an RT-qPCR assay. ^****^ *P* < 0.0001 and ^****^ *P* < 0.0001 as compared with shNC or pcDNA, individually.

Supplementary Figure 2
**(A)** The m^6^A site of SNHG1 was displayed by UCSC Genome Browser (http://genome.ucsc.edu/). **(b, Cc)** The expression of RBM15 mRNA after transfection of specific shRNAs targeted RBM15 was detected by an RT-qPCR assay. ^***^ *P* < 0.001 and ^****^ *P* < 0.0001 as compared with shNC. **(d)** The expression of RBM15 protein after transfection of specific shRNAs targeted RBM15 was detected by a western blot assay. ^****^ *P* < 0.0001 and ^####^ *P* < 0.0001 as compared with shNC, respectively.

Supplementary Figure 3
**(a)** The subcellular localization of SNHG1 was presented by an RNA-FISH assay. **(b)** Genomic location of SNHG1 and its neighboring genes was identified by UCSC. **(c–e)** The correlation between SNHG1 and SLC3A2 in TARGET-OS, GSE33458 and GSE87437 was determined by a spearman correlation analysis.

## References

[1] Valery PC Laversanne M Bray F. Bone cancer incidence by morphological subtype: a global assessment. Cancer causes control: CCC. (2015) 26:1127–39. doi: 10.1007/s10552-015-0607-3 26054913

[2] Mirabello L Troisi RJ Savage SA. Osteosarcoma incidence and survival rates from 1973 to 2004: data from the Surveillance, Epidemiology, and End Results Program. Cancer. (2009) 115:1531–43. doi: 10.1002/cncr.24121 PMC2813207 19197972

[3] Beird HC Bielack SS Flanagan AM Gill J. Osteosarcoma. Nat Rev Dis primers. (2022) 8:77. doi: 10.1038/s41572-022-00409-y 36481668

[4] Wang T Kong S Tao M Ju S. The potential role of RNA N6-methyladenosine in Cancer progression. Mol cancer. (2020) 19:88. doi: 10.1186/s12943-020-01204-7 32398132 PMC7216508

[5] Zeng C Huang W. Roles of METTL3 in cancer: mechanisms and therapeutic targeting. J Hematol oncology. (2020) 13:117. doi: 10.1186/s13045-020-00951-w PMC7457244 32854717

[6] Li T Hu PS Zuo Z Lin JF Li X Wu QN. METTL3 facilitates tumor progression via an m(6)A-IGF2BP2-dependent mechanism in colorectal carcinoma. Mol cancer. (2019) 18:112. doi: 10.1186/s12943-019-1038-7 31230592 PMC6589893

[7] Wang Q Chen C Ding Q Zhao Y Wang Z Chen J. METTL3-mediated m(6)A modification of HDGF mRNA promotes gastric cancer progression and has prognostic significance. Gut. (2020) 69:1193–205. doi: 10.1136/gutjnl-2019-319639 31582403

[8] Wang Q Guo X Li L Gao Z Su X Ji M. N(6)-methyladenosine METTL3 promotes cervical cancer tumorigenesis and Warburg effect through YTHDF1/HK2 modification. Mol cancer. (2020) 11:911. doi: 10.1038/s41419-020-03071-y PMC7585578 33099572

[9] Liu HT Zou YX Zhu WJ Sen-Liu Zhang GH Ma RR. lncRNA THAP7-AS1, transcriptionally activated by SP1 and post-transcriptionally stabilized by METTL3-mediated m6A modification, exerts oncogenic properties by improving CUL4B entry into the nucleus. Cell Death differentiation. (2022) 29:627–41. doi: 10.1038/s41418-021-00879-9 PMC8901790 34608273

[10] Chang YZ Chai RC Pang B Chang X An SY Zhang KN. METTL3 enhances the stability of MALAT1 with the assistance of HuR via m6A modification and activates NF-κB to promote the Malignant progression of IDH-wildtype glioma. Cancer letters. (2021) 511:36–46. doi: 10.1016/j.canlet.2021.04.020 33933553

[11] Liu J Yuan JF Wang YZ. METTL3-stabilized lncRNA SNHG7 accelerates glycolysis in prostate cancer via SRSF1/c-Myc axis. Cell Death differentiation. (2022) 416:113149. doi: 10.1016/j.yexcr.2022.113149 35405116

[12] Zhou X Yang Y Li Y Liang G Kang D Zhou B. METTL3 Contributes to Osteosarcoma Progression by Increasing DANCR mRNA Stability via m6A Modification. Front Cell Dev Biol. (2021) 9:784719. doi: 10.3389/fcell.2021.784719 35096816 PMC8790117

[13] Thin KZ Tu JC Raveendran S. Long non-coding SNHG1 in cancer. Clinica chimica acta; Int J Clin Chem. (2019) 494:38–47. doi: 10.1016/j.cca.2019.03.002 30849309

[14] Huang L Jiang X Wang Z Zhong X Tai S Cui Y. Small nucleolar RNA host gene 1: A new biomarker and therapeutic target for cancers. Pathology Res Pract. (2018) 214:1247–52. doi: 10.1016/j.prp.2018.07.033 30107989

[15] Deng R Zhang J Chen J. lncRNA SNHG1 negatively regulates miRNA−101−3p to enhance the expression of ROCK1 and promote cell proliferation, migration and invasion in osteosarcoma. Int J Mol Med. (2019) 43:1157–66. doi: 10.3892/ijmm.2018.4039 PMC6365036 30592267

[16] Li Z Wang X Liang S. Long non-coding RNA small nucleolar RNA host gene 1 knockdown suppresses the proliferation, migration and invasion of osteosarcoma cells by regulating microRNA-424-5p/FGF2 *in vitro* . Exp Ther Med. (2021) 21:325. doi: 10.3892/etm.2021.9756 33732298 PMC7903380

[17] Wang J Cao L Wu J Wang Q. Long non-coding RNA SNHG1 regulates NOB1 expression by sponging miR-326 and promotes tumorigenesis in osteosarcoma. Int J Oncol. (2018) 52:77–88. doi: 10.3892/ijo.2017.4187 29115574 PMC5743365

[18] Liu Q Luo J Wang H Zhang L Jin G. SNHG1 functions as an oncogenic lncRNA and promotes osteosarcoma progression by up-regulating S100A6 via miR-493-5p. Acta Biochim Biophys Sin. (2022) 54:137–47. doi: 10.3724/abbs.2021014 PMC9909214 35130629

[19] Jiang Z Jiang C Fang J. Up-regulated lnc-SNHG1 contributes to osteosarcoma progression through sequestration of miR-577 and activation of WNT2B/Wnt/β-catenin pathway. Biochem Biophys Res Commun. (2018) 495:238–45. doi: 10.1016/j.bbrc.2017.11.012 29108989

[20] Statello L Guo CJ Chen LL. Gene regulation by long non-coding RNAs and its biological functions. Nat Rev Mol Cell Biol. (2021) 22:96–118. doi: 10.1038/s41580-020-00315-9 33353982 PMC7754182

[21] Cheng F Wang L Yi S Liu G. Long non-coding RNA SNHG1/microRNA-195-5p/Yes-associated protein axis affects the proliferation and metastasis of gastric cancer via the Hippo signaling pathway. Funct Integr Genomics. (2022) 22:1043–55. doi: 10.1007/s10142-022-00876-2 35819551

[22] Yang H Gong C Wu Y Xie X Chen Y Li Z. LncRNA SNHG1 facilitates colorectal cancer cells metastasis by recruiting HNRNPD protein to stabilize SERPINA3 mRNA. Cancer Lett. (2024) 604:217217. doi: 10.1016/j.canlet.2024.217217 39233042

[23] Zhang M Wang W Li T Yu X Zhu Y Ding F. Long noncoding RNA SNHG1 predicts a poor prognosis and promotes hepatocellular carcinoma tumorigenesis. BioMed Pharmacother. (2016) 80:73–9. doi: 10.1016/j.biopha.2016.02.036 27133041

[24] Zhang HY Yang W Zheng FS Wang YB Lu JB. Long non-coding RNA SNHG1 regulates zinc finger E-box binding homeobox 1 expression by interacting with TAp63 and promotes cell metastasis and invasion in Lung squamous cell carcinoma. BioMed Pharmacother. (2017) 90:650–8. doi: 10.1016/j.biopha.2017.03.104 28415044

[25] Sun Y Wei G Luo H Wu W Skogerbø G Luo J. The long noncoding RNA SNHG1 promotes tumor growth through regulating transcription of both local and distal genes. Oncogene. (2017) 36:6774–83. doi: 10.1038/onc.2017.286 28825722

[26] Chandrashekar DS Karthikeyan SK Korla PK Patel H Shovon AR Athar M. UALCAN: An update to the integrated cancer data analysis platform. Neoplasia. (2022) 25:18–27. doi: 10.1016/j.neo.2022.01.001 35078134 PMC8788199

[27] Nan A Chen L Zhang N Jia Y Li X Zhou H. Circular RNA circNOL10 inhibits lung cancer development by promoting SCLM1-mediated transcriptional regulation of the humanin polypeptide family. Advanced Sci (Weinheim Baden-Wurttemberg Germany). (2019) 6:1800654. doi: 10.1002/advs.201800654 PMC6343086 30693177

[28] Zeng K Chen X Xu M Liu X Hu X Xu T. CircHIPK3 promotes colorectal cancer growth and metastasis by sponging miR-7. Cell Death disease. (2018) 9:417. doi: 10.1038/s41419-018-0454-8 29549306 PMC5856798

[29] Wang Y Yang T Zhang Z Lu M Zhao W Zeng X. Long non-coding RNA TUG1 promotes migration and invasion by acting as a ceRNA of miR-335-5p in osteosarcoma cells. Cancer Sci. (2017) 108:859–67. doi: 10.1111/cas.13201 PMC5448616 28205334

[30] Cheng Z Yu C Cui S Wang H Jin H Wang C. circTP63 functions as a ceRNA to promote lung squamous cell carcinoma progression by upregulating FOXM1. Nature communications. (2019) 10:3200. doi: 10.1038/s41467-019-11162-4 PMC6642174 31324812

[31] Liu Y Qiu G Luo Y Li S Xu Y Zhang Y. Circular RNA ROCK1, a novel circRNA, suppresses osteosarcoma proliferation and migration via altering the miR-532-5p/PTEN axis. Exp Mol medicine. (2022) 54:1024–37. doi: 10.1038/s12276-022-00806-z PMC9356001 35879346

[32] Seo JS Chua NH. Identification of long noncoding RNA-protein interactions through *in vitro* RNA pull-down assay with plant nuclear extracts. Methods Mol Biol (Clifton NJ). (2019) 1933:279–88. doi: 10.1007/978-1-4939-9045-0_17 30945192

[33] Zhou C Wang D. TGFB2-AS1 inhibits triple-negative breast cancer progression via interaction with SMARCA4 and regulating its targets TGFB2 and SOX2. Proc Natl Acad Sci United States America. (2022) 119:e2117988119. doi: 10.1073/pnas.2117988119 PMC9522332 36126099

[34] Wang Y Zeng X Wang N Zhao W Zhang X Teng S. Long noncoding RNA DANCR, working as a competitive endogenous RNA, promotes ROCK1-mediated proliferation and metastasis via decoying of miR-335-5p and miR-1972 in osteosarcoma. Mol Cancer. (2018) 17:89. doi: 10.1186/s12943-018-0837-6 29753317 PMC5948795

[35] Yang J Li Q Noureen N Fang Y Kurmasheva R Houghton PJ. PCAT: an integrated portal for genomic and preclinical testing data of pediatric cancer patient-derived xenograft models. Nucleic Acids Res. (2021) 49:D1321–d1327. doi: 10.1093/nar/gkaa698 32810235 PMC7778893

[36] Dai D Wang H Zhu L Jin H Wang X. N6-methyladenosine links RNA metabolism to cancer progression. Cell Death Dis. (2018) 9:124. doi: 10.1038/s41419-017-0129-x 29374143 PMC5833385

[37] He Y Wang W Xu X Yang B Yu X Wu Y. Mettl3 inhibits the apoptosis and autophagy of chondrocytes in inflammation through mediating Bcl2 stability via Ythdf1-mediated m(6)A modification. Nucleic Acids Res. (2022) 154:116182. doi: 10.1016/j.bone.2021.116182 34530171

[38] Chen L Zhang C Ma W. METTL3-mediated m6A modification stabilizes TERRA and maintains telomere stability. Nucleic Acids Res. (2022) 50:11619–34. doi: 10.1093/nar/gkac1027 PMC9723618 36399511

[39] Zhou Y Zeng P Li YH Zhang Z Cui Q. SRAMP: prediction of mammalian N6-methyladenosine (m6A) sites based on sequence-derived features. Nucleic Acids Res. (2016) 44:e91. doi: 10.1093/nar/gkw104 26896799 PMC4889921

[40] Patil DP Chen CK Pickering BF Chow A Jackson C Guttman M. m(6)A RNA methylation promotes XIST-mediated transcriptional repression. Nature. (2016) 537:369–73. doi: 10.1038/nature19342 PMC5509218 27602518

[41] Qin Y Hou Y Liu S Zhu P Wan X Zhao M. A Novel Long Non-Coding RNA lnc030 Maintains Breast Cancer Stem Cell Stemness by Stabilizing SQLE mRNA and Increasing Cholesterol Synthesis. Advanced science (Weinheim, Baden-Wurttemberg, Germany). (2021) 8:2002232. doi: 10.1002/advs.202002232 33511005 PMC7816696

[42] Xuan Y Wang Y. Long non-coding RNA SNHG3 promotes progression of gastric cancer by regulating neighboring MED18 gene methylation. Cell Death disease. (2019) 10:694. doi: 10.1038/s41419-019-1940-3 31534128 PMC6751301

[43] Gorthi A Romero JC Loranc E Cao L Lawrence LA Goodale E. EWS-FLI1 increases transcription to cause R-loops and block BRCA1 repair in Ewing sarcoma. Nature. (2018) 555:387–91. doi: 10.1038/nature25748 PMC6318124 29513652

[44] Wang P Doxtader KA Nam Y. Structural basis for cooperative function of mettl3 and mettl14 methyltransferases. Mol Cell. (2016) 63:306–17. doi: 10.1016/j.molcel.2016.05.041 PMC4958592 27373337

[45] Zhou H Yin K Zhang Y Tian J Wang S. The RNA m6A writer METTL14 in cancers: Roles, structures, and applications. Biochim Biophys Acta Rev cancer. (2021) 1876:188609. doi: 10.1016/j.bbcan.2021.188609 34375716

[46] Wang X Lu Z Gomez A Hon GC Yue Y Han D. N6-methyladenosine-dependent regulation of messenger RNA stability. Nature. (2014) 505:117–20. doi: 10.1038/nature12730 PMC3877715 24284625

[47] Huang H Weng H Sun W Qin X Shi H Wu H. Recognition of RNA N(6)-methyladenosine by IGF2BP proteins enhances mRNA stability and translation. Nat Cell Biol. (2018) 20:285–95. doi: 10.1038/s41556-018-0045-z PMC5826585 29476152

[48] Zheng W Dong X Dong Y Wang S Jiang H Zhang M. (2019). Multiple Functions and Mechanisms Underlying the Role of METTL3 in Human Cancers. Frontiers in Oncol, 9:1403. doi: 10.3389/fonc.2019.01403 PMC6920212 31921660

[49] Li Y Weng H Chen XY Zhang J Zhu JS. The role of m(6)A RNA methylation in human cancer. J Hematol oncology. (2019) 18:103. doi: 10.1186/s12943-019-1033-z PMC6540575 31142332

[50] Miao W Chen J Jia L Ma J Song D. The m6A methyltransferase METTL3 promotes osteosarcoma progression by regulating the m6A level of LEF1. Biochem Biophys Res Commun. (2019) 516:719–25. doi: 10.1016/j.bbrc.2019.06.128 31253399

[51] Wang J Wang W Huang X Cao J Hou S Ni X. m6A-dependent upregulation of TRAF6 by METTL3 is associated with metastatic osteosarcoma. J Bone oncology. (2022) 32:100411. doi: 10.1016/j.jbo.2022.100411 PMC8802048 35145841

[52] Jiang R Dai Z Wu J Ji S Sun Y Yang W. METTL3 stabilizes HDAC5 mRNA in an m(6)A-dependent manner to facilitate Malignant proliferation of osteosarcoma cells. Cell Death discovery. (2022) 8:179. doi: 10.1038/s41420-022-00926-5 35396379 PMC8993827

[53] Wang X Feng J Xue Y Guan Z Zhang D Liu Z. Structural basis of N(6)-adenosine methylation by the METTL3-METTL14 complex. Nature. (2016) 534:575–8. doi: 10.1038/nature18298 27281194

[54] Ping XL Sun BF Wang L Xiao W Yang X Wang WJ. Mammalian WTAP is a regulatory subunit of the RNA N6-methyladenosine methyltransferase. Cell Res. (2014) 24:177–89. doi: 10.1038/cr.2014.3 PMC3915904 24407421

[55] Yue Y Liu J Cui X Cao J Luo G. VIRMA mediates preferential m(6)A mRNA methylation in 3’UTR and near stop codon and associates with alternative polyadenylation. Cell discovery. (2018) 4:10. doi: 10.1038/s41421-018-0019-0 29507755 PMC5826926

[56] Wen J Lv R Ma H Shen H He C Wang J. Zc3h13 regulates nuclear RNA m(6)A methylation and mouse embryonic stem cell self-renewal. Mol Cell. (2018) 69:1028–1038.e6. doi: 10.1016/j.molcel.2018.02.015 29547716 PMC5858226

[57] Salmena L Poliseno L Tay Y Kats L Pandolfi PP. A ceRNA hypothesis: the Rosetta Stone of a hidden RNA language? Cell. (2011) 146:353–8. doi: 10.1016/j.cell.2011.07.014 PMC3235919 21802130

[58] Tan X Chen WB Lv DJ Yang TW Wu KH Zou LB. LncRNA SNHG1 and RNA binding protein hnRNPL form a complex and coregulate CDH1 to boost the growth and metastasis of prostate cancer. Cell Death disease. (2021) 12:138. doi: 10.1038/s41419-021-03413-4 33542227 PMC7862296

[59] Yang TW Sahu D Chang YW Hsu CL Hsieh CH. RNA-binding proteomics reveals MATR3 interacting with lncRNA SNHG1 to enhance neuroblastoma progression. J Proteome Res. (2019) 18:406–16. doi: 10.1021/acs.jproteome.8b00693 30516047

[60] Wu X Song P Wang S Qian Z Ying J Gao S. A pan-cancer analysis of the oncogenic role of WD repeat domain 74 in multiple tumors. Front Genet. (2022) 13:860940. doi: 10.3389/fgene.2022.860940 35559034 PMC9086290

[61] Li Y Chen F Shen W Li B Xiang R Qu L. WDR74 induces nuclear β-catenin accumulation and activates Wnt-responsive genes to promote lung cancer growth and metastasis. Cancer letters. (2020) 471:103–15. doi: 10.1016/j.canlet.2019.12.011 31838084

[62] Li Y Zhou Y Li B Chen F Shen W Lu Y. WDR74 modulates melanoma tumorigenesis and metastasis through the RPL5-MDM2-p53 pathway. Oncogene. (2020) 39:2741–55. doi: 10.1038/s41388-020-1179-6 32005977

[63] Cai Z Mei Y Jiang X Shi X. WDR74 promotes proliferation and metastasis in colorectal cancer cells through regulating the Wnt/β-catenin signaling pathway. Open Life Sci. (2021) 16:920–9. doi: 10.1515/biol-2021-0096 PMC8422980 34553072

[64] Paronetto MP. Ewing sarcoma protein: a key player in human cancer. Int J Cell Biol. (2013) 2013:642853. doi: 10.1155/2013/642853 24082883 PMC3776376

[65] Lee J Nguyen PT Shim HS Hyeon SJ Im H Choi MH. EWSR1, a multifunctional protein, regulates cellular function and aging via genetic and epigenetic pathways. Biochim Biophys Acta Mol basis disease. (2019) 1865:1938–45. doi: 10.1016/j.bbadis.2018.10.042 PMC6527469 30481590

[66] Luo Y Blechingberg J Fernandes AM Li S Fryland T Børglum AD. EWS and FUS bind a subset of transcribed genes encoding proteins enriched in RNA regulatory functions. Mol Cancer Ther. (2015) 16:929. doi: 10.1186/s12864-015-2125-9 PMC4647676 26573619

[67] Heisey DAR Jacob S Lochmann TL Kurupi R Ghotra MS Calbert ML. Pharmaceutical interference of the EWS-FLI1-driven transcriptome by cotargeting H3K27ac and RNA polymerase activity in ewing sarcoma. Mol Cancer Ther. (2021) 20:1868–79. doi: 10.1158/1535-7163.mct-20-0489 34315769

