# Supplementary material for: METTL3-mediated SNHG1 m6A modification promotes proliferation and migration through transcriptional regulation of WDR74 in osteosarcoma
Source: Front Oncol. 2025 May 29;15:1529657. doi: 10.3389/fonc.2025.1529657 (PMC12159053; doi:10.3389/fonc.2025.1529657)
Supplement: Supplementary file 1 [file Table1.docx]

**Supplementary table 1. Primer and oligonucleotide sequences used in this study**

| **Gene** | **Primer (sense)** | **Primer (antisense)** |
| --- | --- | --- |
| METTL3 | TTGTCTCCAACCTTCCGTAGT | CCAGATCAGAGAGGTGGTGTAG |
| SNHG1 | GCCAGCACCTTCTCTCTAAAGC | GTCCTCCAAGACAGATTCCATTTT |
| WDR74 | AACGAGACGGAGAAAGAAGAAGCG | CGCTTCTTCTTTCTCCGTCTCGT |
| RBM15 | ACGACCCGCAACAATGAAG | GGAAGTCGAGTCCTCACCAC |
| GAPDH mRNA | GGAGCGAGATCCCTCCAAAAT | GGCTGTTGTCATACTTCTCATGG |
| U6 RT | CGAGCACAGAATCGCTTCACGAATTTGCGTGTCAT | |
| U6 | CTCGCTTCGGCAGCACA | AACGCTTCACGAATTTGCGT |
| **siRNAs/shRNAs/oligos** | **Sense** | **Antisense** |
| shNC | CGCGGATTCTGGATTAGTGCGTCTAGCTTCTCGAGAAGCTAGACGCACTAATCCAGAATCTTTTTG | GCCAAAAAGATTCTGGATTAGTGCGTCTAGCTTCTCGAGAAGCTAGACGCACTAATCCAGAATC |
| shMETTL3-1 | CGCGGCAAGAATTCTGTGACTATGGCTCGAGCCATAGTCACAGAATTCTTGCTTTTTG | GCCAAAAAGCAAGAATTCTGTGACTATGGCTCGAGCCATAGTCACAGAATTCTTGC |
| shMETTL3-2 | CGCGGCTGCACTTCAGACGAATTATCTCGAGATAATTCGTCTGAAGTGCAGCTTTTTG | GCCAAAAAGCTGCACTTCAGACGAATTATCTCGAGATAATTCGTCTGAAGTGCAGC |
| shSNHG1-1 | CGCGGCAGACACAGATTAAGACACTCTCGAGAGTGTCTTAATCTGTGTCTGCTTTTTG | GCCAAAAAGCAGACACAGATTAAGACACTCTCGAGAGTGTCTTAATCTGTGTCTGC |
| shSNHG1-2 | CGCGGGAGCCAATGAAACAGCAGTTCTCGAGAACTGCTGTTTCATTGGCTCCTTTTTG | GCCAAAAAGGAGCCAATGAAACAGCAGTTCTCGAGAACTGCTGTTTCATTGGCTCC |
| siNC | CAUGUACGUGUUUAGGGUCGACAUU | AAUGUCGACCCUAAACACGUACAUG |
| siWDR74 | CAUCACAUGUGUGGAUUCUGGGAUU | AAUCCCAGAAUCCACACAUGUGAUG |
| siEWSR1 | GGGCAACAA AGCUAUGGAA | UUCCAUAGCUUUGUUGCCC |
| shEWSR1-1 | CGCGGGGCAACAAAGCTATGGAACCCTCGAGGGTTCCATAGCTTTGTTGCCCTTTTTG | GCCAAAAAGGGCAACAAAGCTATGGAACCCTCGAGGGTTCCATAGCTTTGTTGCCC |
| shEWSR1-2 | CGCGGCGATGCCACAGTGTCCTATGCTCGAGCATAGGACACTGTGGCATCGCTTTTTG | GCCAAAAAGCGATGCCACAGTGTCCTATGCTCGAGCATAGGACACTGTGGCATCGC |
| shRBM15-1 | CGCGGCCTGTTTCATGAGTTCAAACCTCGAGGTTTGAACTCATGAAACAGGCTTTTTG | GCCAAAAAGCCTGTTTCATGAGTTCAAACCTCGAGGTTTGAACTCATGAAACAGGC |
| shRBM15-2 | CGCGGCATACAGTCTTGAGCCAAGGCTCGAGCCTTGGCTCAAGACTGTATGCTTTTTG | GCCAAAAAGCATACAGTCTTGAGCCAAGGCTCGAGCCTTGGCTCAAGACTGTATGC |
| oeMETTL3 | ATAGCTAGCAT ATGTCGGACACGTGG | ATAGCGGCCGCCTATAAATTCTTAGGTTTAGAGATGATAC |
