# Supplementary material for: METTL3-mediated SNHG1 m6A modification promotes proliferation and migration through transcriptional regulation of WDR74 in osteosarcoma
Source: Front Oncol. 2025 May 29;15:1529657. doi: 10.3389/fonc.2025.1529657 (PMC12159053; doi:10.3389/fonc.2025.1529657)
Supplement: Supplementary file 2 [file Table2.docx]

**Supplementary table 2. Correlation between METTL3 and certain OS-related lncRNAs in TCGA and GEO datasets**

| **Gene name** | **METTL3** | | | | | |
| --- | --- | --- | --- | --- | --- | --- |
|  | **TCGA*** | | **GSE87437** | | **GSE33458** | |
|  | **r value** | ***p* value** | **r value** | ***p* value** | **r value** | ***p* value** |
| DANCR | 0.048 | 0.66 | 0.6575 | 0.0012 | 0.8989 | <0.0001 |
| TUG1 | 0.34 | 0.0015 | 0.2894 | 0.0024 | 0.8983 | <0.0001 |
| ***SNHG1*** | 0.32 | ***0.0027*** | 0.4688 | ***0.0321*** | 0.9438 | ***<0.0001*** |
| UCA1 | 0.071 | 0.52 | -0.2310 | 0.3138 | -0.6869 | 0.0016 |
| BCAR4 | 0.12 | 0.26 | 0.2184 | 0.3415 | 0.2308 | 0.3569 |
| SNHG5 | 0.27 | 0.011 | -0.1243 | 0.5916 | 0.8456 | <0.0001 |
| TUSC7 | 0.023 | 0.83 | -0.5461 | 0.0104 | -0.8519 | <0.0001 |
| MALAT1 | -0.13 | 0.24 | -0.2094 | 0.3623 | 0.8044 | <0.0001 |
| MEG3 | 0.082 | 0.45 | - | - | - | - |
| GAS5 | 0.18 | 0.087 | 0.0546 | 0.8141 | 0.464758002 | 0.052 |

*An online web-tool GEPIA2 was used to analyze the correlation between METTL3 and certain osteosarcoma-related lncRNAs in TCGA.
