# Supplementary material for: METTL3-mediated SNHG1 m6A modification promotes proliferation and migration through transcriptional regulation of WDR74 in osteosarcoma
Source: Front Oncol. 2025 May 29;15:1529657. doi: 10.3389/fonc.2025.1529657 (PMC12159053; doi:10.3389/fonc.2025.1529657)
Supplement: Supplementary file 3 [file Table3.docx]

**Supplementary table 3. The sequences of WDR74 promoter region used in this work.**

WDR promoter sequences:

From 62841809 to 62843809

>NC_000011.10:c62843809-62841809 Homo sapiens chromosome 11, GRCh38.p14 Primary Assembly

AATGGGCTAAAATCAAGGTGTAATGACAGGGCTGCATTGCTTTCTGGAGGCTGCATGGGAGAATCTATTTCCTTGCTTTTTCCAACTTCTAGCTGCTGCCCACAACTCCCTGGCTGATGACTCTCTTCCATCTGCAGAGCAAGCCCTGGCTGCTCAAATCTTCCTCAGGTTGCATCACTCAGACTCTCCTGCCTCCCTCTACCCCTTATAAAGACATTTGTGATTACTCTGAACCCACCCAGAAAATCCAGGATAAATCTCGCCGTCTCAAAATCCTTAATTTAATAATATCTGCAAAATACCTTTTGCTATGTAAGGTAACACATTCAGAGGTTCTCACTGACCATCTCTGGGGTGGGGAGGGCATTATTCTGCTTATTATCCTCTTTTTTTTTTTTTTTTTTTTGAGACAGAGTCTCGCTCTGTCACCCAGGCTGGAGTGTAGTGTCATGATCTCAGATCACTGCAACCTCTGCCTCCCAGGTTCAAGCGATTCTCATGCCTCAGCCTCCCAAGTAGCTGGGACTACAGGCATGCACCATCATGCCTGGCTACTTTTTGTATTTTCAGTAGAGACAGGGTTTTGCCATGTTGCCCAGGCTGGTCTCGAACTCCTGGCCTCAAGTAATCCACCCGCCTTGGCCTCCCGAAGTGTTGGGATTACAGGTGTGAGCCACCACGCCCGGCCCCCTGCCTACTACTCTCTTGATGTGGCACCTCTCTGAAGGCTCTGGGTTTACTTATGAGAACTTGTAGAAATTTCTTTTGTTTATTAATACCAGTGTCATAAAATCATAGATTATCAGAAGCTTTGCCGGTTGACATTTTATCAGTGAGAATGTTAACGCCCCACTCCTGCCTGGAGGATCTCAGCCATGCAAGACGCTTTGATGCAAAGACACCGCCTCTATTTCCAACCCAGGTTAAGGGGTTTCCAACAGAACGATCCACGTTTATATTAATAGAGCTTCCAAGAAAGCAAGACCTTAAGTCCCTTCTCAGCCAGGGCCTCATATTAATCCCTTTCATTCTGGCGAGCTTGCTTTGATCGATTAAAAAAAAATCGCTCCATTTCAATTTTACCTACACTCTCCTCCTCCTCCTTCGCCCCAGTCCTCTAACGACCCTGCCTCTGCCTTCGAGGAGAGGCCCTGTGGTTTCTCGGTCGGACTTCCGGGTGGGATGCCTTGCCGCGTTGCTGGTCGGCCTGAAAGTAACTCTAAAGAGTGCGTGGGTCGGAGGACATCGGGGCTGAGGCGTCAGCACCTTTCTCCCACCGCGGGATCCCGGGTTTGAGGATCAGGAGCCTCTGAAATGGCTTTCTCAGGGCATCTTTCCCTCTCAGTGAAGGGCCAGATGTTTGCTGGCGGACAGTACAAATGCAAGGTGCGGGTCGTGCCTCGCCCCTATGTCCTCAGTTCTCACAAACTCCGGAGAGTGAGTGTCACCATTTCCCCCGTTTAAATGGGGAGGAAACAAGAGCCTAAATACCAAGCCCAGGCCATTAAGTCAGGAACTGGAGAGCAGGGAGCCCAACCTCCTTTTTTGGACTCCAAAACCTGTGCTTTGACACCGCGCCGCGGCTTCTCCCAAGAAAGGTCCCGGAGAGGGGCGCCGCCACCTGCAGCACTGTCCCGCCGCCCCCTGGAAAGCCGAAAAACCCCTAGGATGGGAGACTAACGCCACAATCGGGGAAAAGGAGGCCACGCGGGCATTGAAATCACGACTGGTGTGTGGACCAATCCCAGGATCCTAAAAAAGCGAAAGGGGCGGGTTTATGCAAAACAAACTGAGCAGGGAGCAGGCGGCGGAAGAAGTAGAGGACGTTTAAATAGGGCTGTTTCCAAAATATAGCCACTACTATAAAGAAAGAATAATGAAAACTGTGTTTCGGAATTATAATGTATTGGGAGATAATTTAACATTTAGTGCCTGGATAGTTACCATAACTGGTTGGAAGATGGGAAGGATAAGGCCGCCGAGGCGACCGAAGTAAAGGTA
