# Supplementary figures and images for: METTL3-mediated SNHG1 m6A modification promotes proliferation and migration through transcriptional regulation of WDR74 in osteosarcoma
Source: Front Oncol. 2025 May 29;15:1529657. doi: 10.3389/fonc.2025.1529657 (PMC12159053; doi:10.3389/fonc.2025.1529657)

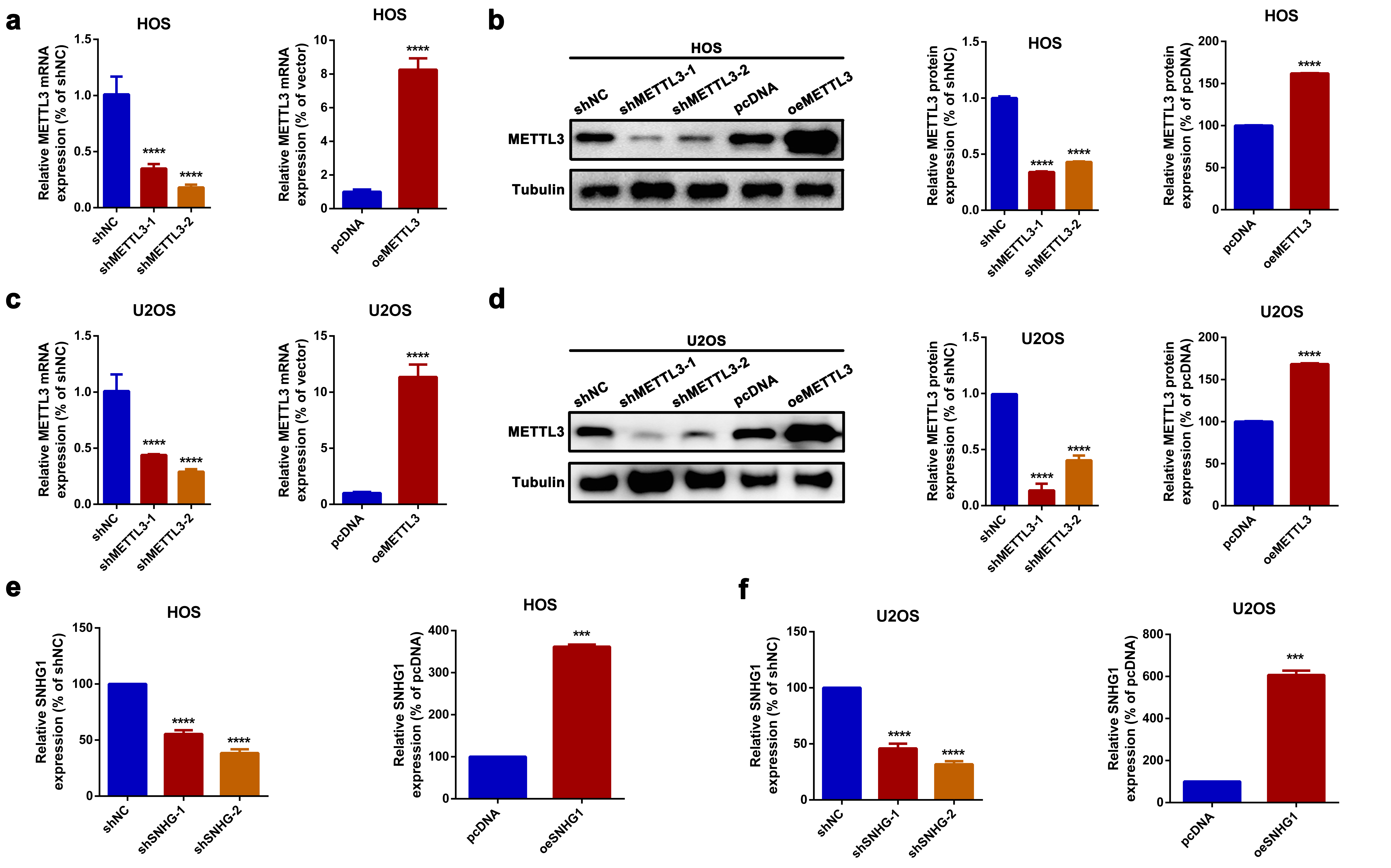

Supplement: Supplementary Figure 1 — (a, c) Expression of METTL3 mRNA after transfection of specific shRNAs targeted METTL3 or stable overexpression plasmids carrying METTL3 in HOS and U2OS cells was detected by an RT-qPCR assay. **** P < 0.0001 and **** P < 0.0001 as compared with shNC or pcDNA, individually. (b, d) Expression of METTL3 protein after transfection of specific shRNAs targeted METTL3 or stable overexpression plasmids carrying METTL3 in HOS or U2OS cells was detected by an RT-qPCR assay. **** P < 0.0001 and **** P < 0.0001 as compared with shNC or pcDNA, separately. (e, f) Expression of SNHG1 after transfection of specific shRNAs targeted SNHG1 or stable overexpression plasmids carrying SNHG1 in HOS or U2OS cells was detected by an RT-qPCR assay. **** P < 0.0001 and **** P < 0.0001 as compared with shNC or pcDNA, individually. [file Image1.jpeg]

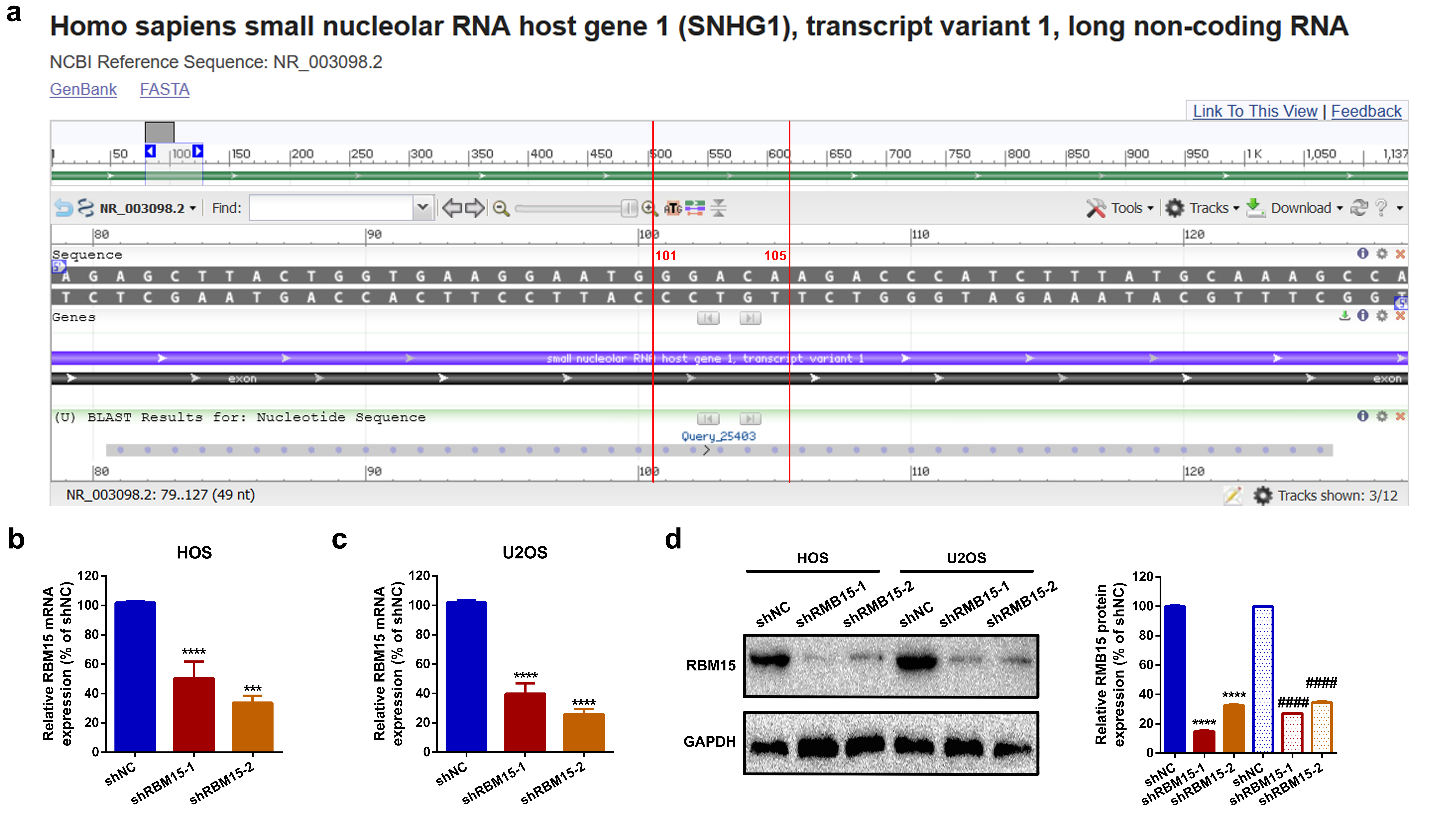

Supplement: Supplementary Figure 2 — (A) The m6A site of SNHG1 was displayed by UCSC Genome Browser (http://genome.ucsc.edu/). (b, Cc) The expression of RBM15 mRNA after transfection of specific shRNAs targeted RBM15 was detected by an RT-qPCR assay. *** P < 0.001 and **** P < 0.0001 as compared with shNC. (d) The expression of RBM15 protein after transfection of specific shRNAs targeted RBM15 was detected by a western blot assay. **** P < 0.0001 and #### P < 0.0001 as compared with shNC, respectively. [file Image2.jpeg]

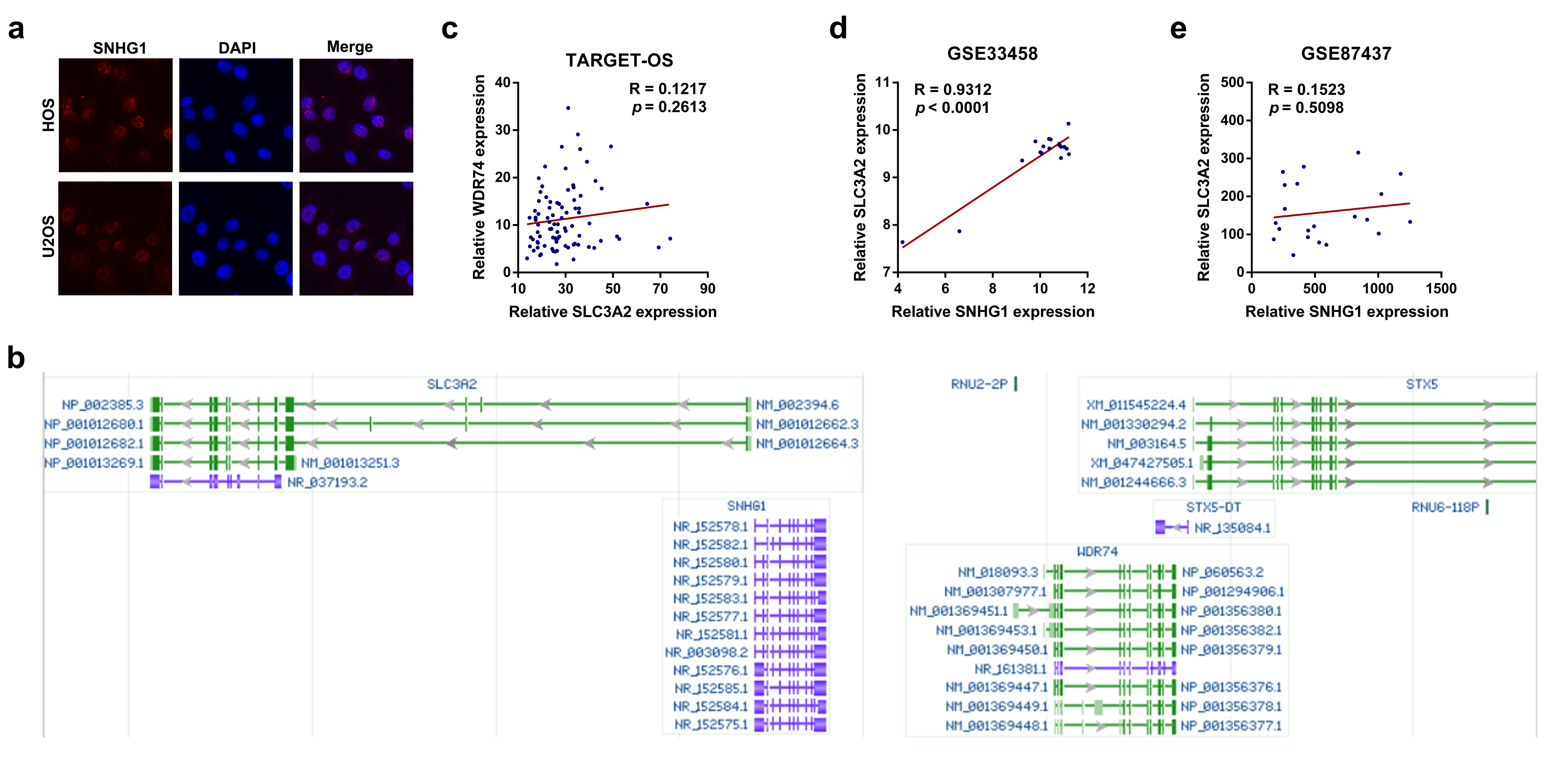

Supplement: Supplementary Figure 3 — (a) The subcellular localization of SNHG1 was presented by an RNA-FISH assay. (b) Genomic location of SNHG1 and its neighboring genes was identified by UCSC. (c–e) The correlation between SNHG1 and SLC3A2 in TARGET-OS, GSE33458 and GSE87437 was determined by a spearman correlation analysis. [file Image3.jpeg]
